# Can Large Language Models Generate Useful Linguistic Corpora?: A Case Study of the Word Frequency Effect in Young German Readers

**DOI:** 10.1162/OPMI.a.30

**Published:** 2025-10-29

**Authors:** Job Schepens, Hanna Woloszyn, Nicole Marx, Benjamin Gagl

**Affiliations:** Institute for Digital Humanities, University of Cologne, Cologne, Germany; Self learning systems Lab, University of Cologne, Cologne, Germany; Mercator Institute, University of Cologne, Cologne, Germany

**Keywords:** large language models, linguistic corpus, word frequency effect, lexical decision task

## Abstract

Linguistic corpora are an essential resource in psycholinguistic research. Here, we generate new corpora using large language models (LLMs) and determine their usefulness for estimating the word frequency effect on reading performance, focusing on German children. We prompted three different LLMs to create corpora of children’s stories using the titles of 500 books, mimicking an existing corpus of children’s books (childLex). In Experiment 1, we found that word frequency correlated strongly between childLex and the LLM corpora, despite a lower lexical richness of LLM text. Compared to childLex, we found that the estimated effect size of the LLM-based word frequency effect was lower, but that it explained more variance in reading performance (using reaction times for about 1000 words in a lexical decision task). In Experiment 2, we found that prompting for children-directed text results in word frequency that better fits to child compared to adult reading times, and also that increasing temperature can increase lexical richness. In Experiment 3, we replicated Experiment 1 using two open-weight LLMs. Across all 10 corpora (out of which 9 were LLM-based), we found that corpora with lower lexical richness generally fit better to reaction times. We discuss the potential of this approach, considering the risks associated with utilizing highly complex large language models (LLMs).

## INTRODUCTION

In recent years, Large Language Models (LLMs) have improved quickly, enabling new ways of interaction between humans and computers (Bommasani et al., 2022) across different languages and contexts (Chang et al., 2023; Lai et al., 2023), despite their vastly non-human-like nature (Evanson et al., 2023; Kasneci et al., 2023; Min et al., 2021; Singhal et al., 2023). LLMs are now being used in research contexts, including psycholinguistics, where they have been applied to generate predictors that explain variance in reading behavior and brain activation related to word predictability (Boeve & Bogaerts, 2024; Botch & Finn, 2024; Chandra et al., 2023; Heilbron et al., 2023; Hofmann et al., 2022; Lopes Rego et al., 2024). This context seems promising, given that LLMs are trained on next-word predictability (Tay et al., 2022). Various other psycholinguistics measures have been studied using LLMs, ranging from augmentation (Trott, 2024), to semantic (Caucheteux & King, 2022) and syntactic processing (Desbordes et al., 2023; Fresen et al., 2024). The goodness of fit of LLMs to human reading times seems closely connected to their numbers of parameters (Oh & Schuler, 2023) as well as word frequency in the training data (Oh & Schuler, 2025; Oh et al., 2024). Here, instead of word frequency in training data, we study word frequency in corpora of texts generated by LLMs.

Word frequency plays a central role in word recognition and reading studies, as it has a large effect on both adult and child reading performance (Brysbaert et al., 2018; Brysbaert et al., 2016; Gregorová et al., 2023; Hawelka et al., 2010; Kliegl et al., 2004; Schröter & Schroeder, 2017). The frequency of a word is typically measured by taking large linguistic corpora and counting its occurrences (e.g., see Baayen et al., 1993; Brysbaert et al., 2011; Heister et al., 2011; Schroeder et al., 2015). With ever-expanding corpora, increasingly appropriate word frequency estimates are possible, which may describe the variance in word recognition tasks better than other existing measures (Brysbaert et al., 2011). **In this paper, we investigate whether an LLM corpus can be used to extract a word frequency measure which may better describe the variance in word recognition performance than existing corpora.** The goal of these evaluations is to test if the integration of LLM corpora into psycholinguistic research is reasonable, potentially identifying a way of using LLM corpora for estimating specific word frequency measures for selected groups (in our case, German school children).

We focus on beginning German readers for various reasons. First, we anticipate needing a smaller-sized corpus for this group. Further, it is possible to draw on two openly available datasets for evaluation. (i) childLex (Schroeder et al., 2015) provides a high-quality corpus of 500 books written for children, allowing a direct comparison of LLM corpora with an existing book-based corpus. (ii) DeveL (Schröter & Schroeder, 2017) provides reading performance data for a large set of words based on reaction times in a lexical decision task. We compare book-based and LLM-based word frequency to investigate which measure explains more variance in reaction times in a lexical decision task, which is often used to study reading performance.

The effect of word frequency on reading performance is considered substantial and robust (Brysbaert et al., 2016, 2018) across groups (e.g., Hawelka et al., 2010) and modalities (e.g., Gregorová et al., 2023). The word frequency effect describes that words that often occur (i.e., high-frequency words) are recognized faster and more accurately compared to less common words (i.e., low-frequency words; Adelman et al., 2006; Baayen, 2010; Brysbaert et al., 2016; Gregorová et al., 2023; Hallin & Reuterskiöld, 2018; Lieven, 2010; S. A. McDonald & Shillcock, 2001; Stokes, 2010). To accurately estimate word frequency, the choice of the underlying corpus is highly relevant. Previous studies have collected large numbers of books and newspapers and combined them into corpora for measuring word frequency (e.g., Baayen et al., 1993; Heister et al., 2011). The use and validity of frequency measures depend on the source of the text. For example, Brysbaert and colleagues (Brysbaert et al., 2011, 2018) have shown that word frequency statistics based on television and movie subtitles explain more of the variance in word processing difficulty performance measures such as lexical decision response times than book-based corpora (see also Chilson et al., 2024). The critical comparison here is based on model comparisons utilizing reading performance data; i.e., a regression model involving subtitle-based word frequency measures had a higher model fit than a book-based word frequency measure (see, e.g., Brysbaert et al., 2011). Thus, the corpus used to derive word frequency significantly influences the amount of variance that word frequency can explain (Ferrand et al., 2010; Keuleers et al., 2010; Van Heuven et al., 2014), even when carefully controlling for other essential word characteristics such as orthographic similarity to other words, age in which a word is typically acquired, and word length (Graf et al., 2005).

Only a few linguistic corpora exist that can be used to measure word frequency related to children. These are typically based on children’s books or subtitles for children’s movies (Korochkina et al., 2024; Schroeder et al., 2015; Tellings et al., 2014; Van Heuven et al., 2014). Constructing a text corpus specifically designed for children is challenging for several reasons. The number of materials developed explicitly for children is necessarily smaller than that for adults. Access to a wide range of children’s literature and other resources can be limited due to, for example, questionable validity or even availability of the listed or estimated target age range. Estimating the target age can be problematic, as not all materials explicitly indicate a target age group. Finally, there are only a few available resources for text written by children themselves (see, e.g., Laarmann-Quante et al., 2019). Together, these factors result in a limited and possibly biased set of language materials, which is expected to have implications for studies that use word frequency measures.

In summary, previous studies have shown that text type is important for studying the effects of word frequency on reading performance; however, corpora involving text written for children are scarce. LLMs can potentially help to better understand word frequency effects. However, little is known about the usefulness of LLM text in this regard. Our primary objective is thus to develop a measure of word frequency for children derived from LLM text and evaluate its potential usefulness for estimating word frequency effects.

Our procedure is as follows. First, we generate a corpus of texts that are based on the titles of the books contained in childLex. Then, we compare word frequency between both corpora. Finally, we utilize a large reading performance dataset (DeveL) that includes data from children, adolescents, and adults (Schröter & Schroeder, 2017) to evaluate whether LLM word frequency better fits reaction times than childLex word frequency. This procedure is similar to previously performed evaluations of measures of word frequency (e.g., Brysbaert et al., 2011, 2018). Using reaction times from a lexical decision task as a measure of reading performance is a common choice for studies on word recognition in children (Davies et al., 2017; Monster et al., 2022; van den Boer et al., 2012).

### Strengths and Weaknesses of using LLMs for Psycholinguistic Research

LLMs are computational models with a considerable amount of trainable parameters - in the case of ChatGPT-3.5, 175 billion (Brown et al., 2020). This makes the inner workings opaque, but potent for learning language-related tasks such as next-word or next-sentence prediction with high accuracy (e.g., Devlin et al., 2019). LLMs make use of architectural principles such as transformers and attention heads (Vaswani et al., 2017); see also (Hussain et al., 2024, for an introduction). To be able to produce comprehensible output text, LLMs need to be trained on a vast set of data (Bender et al., 2021).

Several weaknesses of LLMs have been pointed out. Little is known about the minimal training data requirements (Hosseini et al., 2022). LLM training data is not developmentally realistic when compared to human input (Evanson et al., 2023; Feng et al., 2024; Warstadt et al., 2023). Also, the required amounts of computing resources and training data can make it technically challenging to apply interventions or change aspects of the architecture or training procedure, so they are typically fine-tuned using reinforcement learning with human feedback (RLHF) to generate output that aligns with desired outcomes (see Ouyang et al., 2022, for how to remove troubling model outputs). Given the bias in the training data that is typically used, the output of LLMs is biased in favor of Western culture (Atari et al., 2023). Furthermore, for many LLMs, important information is unavailable, as they lack transparency (Frank, 2023b; Liesenfeld et al., 2023). The lack of transparency and the considerable effort required for training these models make reproducing the models a challenging task. This also questions what kind of LLMs should be and should not be used in research or as research objects (Bender et al., 2021; Liesenfeld et al., 2023).

Despite these caveats, LLMs seem to have much potential for studying word frequency (Oh et al., 2024) or other psycholinguistic measures (Martínez et al., 2024). LLMs have likely seen more words (and possibly languages) than any human. Extensive training data in combination with next-word-prediction may be a strength for estimating word frequency compared to traditional measures, since estimating the probability of words is part of how LLMs are optimized. Finally, the output of LLMs is generally flexible. It depends on the model prompt and a set of parameters (for the temperature parameter: higher values lead to greater lexical diversity in the generated text, similar to exploration in humans Momennejad et al., 2023).

What uses can LLMs have in research on language processing? Here, we assume that LLMs can be useful as tools, rather than a theoretical approach to language (e.g., as in Binz et al., 2024). So far, LLMs have been used to expose necessary conditions and qualities of the learning environments for human language acquisition (Trott et al., 2023; Warstadt et al., 2023), predict human brain responses to language (Tuckute et al., 2024), estimate word predictability from sentence context (Chandra et al., 2023; Heilbron et al., 2021; Hofmann et al., 2022), and explain N400 effects (Michaelov et al., 2024). Such efforts emphasize the importance of human benchmarks, which are crucial for evaluating how LLM capacities align with human capabilities. LLMs also deviate from human behavior in interesting ways (Mahowald et al., 2024). For example, LLMs have trouble with language comprehension (Dentella et al., 2024), and LLM optimization is very different from the pressures that shape human performance (McCoy et al., 2023). Regarding child reading research, their large amount of training data and training procedures enables LLMs to represent various linguistic contexts, which may reflect exposure to words in ways that are relevant to word frequency measurement. Here, we explore the latter aspect.

### Present Study

In three studies, we investigate whether and how LLMs can generate corpora for measuring word frequency that are suitable for approximating the lexicon of young German readers. ChildLex (Schroeder et al., 2015), our reference corpus, is based on children’s books. In Experiment 1, we generate a corpus modeled after childLex (Schroeder et al., 2015). To implement this, we prompt the model for child-directed stories based on the book titles included in the original corpus. In Experiment 2, we generate four additional corpora, systematically varying the target audience of the stories using different prompts (i.e., child-directed vs. adult-directed text) and also varying the model parameter responsible for lexical variability in the text output (high vs. low temperature). In Experiment 3, we again generate four new corpora using two open-weight LLMs, as well as manipulating text length (long vs. short).

## EXPERIMENT 1

After generating a corpus of LLM-based text, we evaluate it by comparing resulting word frequency measures to childLex (Schroeder et al., 2015). In the second step, we assess the word frequency measures against reading performance (Schröter & Schroeder, 2017) in younger and older adults by testing which measure best explains the behavioral data.

### Method

#### Model Choice.

Despite reservations about the openness of the model (see, e.g., Hussain et al., 2024; Liesenfeld et al., 2023), we chose to use “GPT-3.5-turbo” for Experiments 1 and 2, which pointed to Snapshot Version 0301 (the first version made available). This is an efficient and optimized version of GPT-3.5, with a likely smaller number of parameters. This off-the-shelf model was usable without any initial training or setup and was stable, state-of-the-art, and affordable. In May 2023, the model showed good performance was easy to handle via an API (i.e., easy to use via Python; find the script here: osf.io, was stable (not the case with the GPT-4 version at the time), and was cost-effective (the pricing at the time of generations was $0.002 / 1K tokens, which was more affordable than $0.06 / 1K tokens for GPT-4). In this context, a token can be a word, part of a word, or punctuation mark. The tokenizer splits the training data into tokens by iteratively combining the most frequently adjacent pairs of tokens until it reaches a pre-specified vocabulary size. The model had a token limit of 4,096 tokens, equivalent to approximately 3,000 words. This limit included both the length of the input prompt and the generated output. The texts that we generated were substantially shorter than texts typically used to estimate word frequency, e.g., full books or films. To account for this length difference, we used the same prompt repeatedly to generate different texts – an interesting option since LLMs generate different texts for each prompt, even when the prompt remains constant. This strategy also ensured a comparable length of text generated for each book title, which could otherwise have biased the results.

#### LLM Prompt Engineering.

Traditionally, word frequency is measured using books. ChildLex uses the texts of 500 popular books written for children in several different age ranges (for details, see (Schroeder et al., 2015). Titles and age ranges include such works as “Karius und Baktus” for children aged 4–6, “King-Kong, das Schulschwein” (King-Kong, the School Pig), for children aged 8–10, and “Der Fluch des Goldes” (The Golden Curse), for children aged 14–17. We decided to use the titles of these books to prompt the LLM in the direction of the themes of these books. Note that we are unaware if the LLM we used had these books as part of its large set of training data, but the likelihood that at least some of them were part of it is high, given the size of the LLM (Y. Liu et al., 2024).

Using these book titles, our prompts had the following structure: *4000Wörter zu **Buchtitel** auf Deutsch für Kinder* (4000 Words on **Booktitle** in German for Children). In case the age range was known, it was added (*im Alter **Altersangabe***; at the age of **age range**), with **Booktitle** and **Altersangabe** changing for every specific book title. We deliberately kept our prompts simple to minimize prompt engineering; future studies could (and should) improve the prompts by, for example, requesting storytelling and narrative elements, providing more context, or providing information about our goal (i.e., estimating word frequency).

Since we kept the temperature set at .5, the text output was balanced between deterministic and random. It turned out that this prompt results on average in 628 words per prompt. For reasons which are unclear, the LLM produces substantially shorter stories than what is asked for in the prompt. It is possible to engineer prompts that result in longer texts, for example, by prompting to divide the story into chapters. Subjectively, the resulting texts often seem to relate to the content of these books. For example, *“Es war ein sonniger Tag im Frühling und Opa Franz war im Garten beschäftigt. Er war gerade dabei, die Blumenbeete zu jäten, als er plötzlich ein seltsames Geräusch hörte. Es klang wie ein Schnauben und ein Fauchen zugleich. Verwundert drehte er sich um und sah etwas, das er zuvor noch nie gesehen hatte. Ein kleiner Drache saβ auf dem Zaun und betrachtete ihn neugierig.”*, which translates to *“It was a sunny day in spring and Grandpa Franz was busy in the garden. He was weeding the flowerbeds when he suddenly heard a strange noise. It sounded like a snort and a hiss at the same time. Puzzled, he turned around and saw something he had never seen before. A little dragon was sitting on the fence, watching him curiously.”* (See repository with all texts here: osf.io)

#### Corpus Design.

We decided to generate texts 20 times for each book title to increase representativeness and saturation (see Barth & Schnell, 2021) and increase the total amount of generated text per book. To implement the repeated text generation, we set the n-parameter (number of prompts per run) to 4 and then ran the prompt five times for all 500 books. We stored the result of every prompt in a separate text file (filename: “Story_” + N + “.txt”, where N represents the number of books on the list). This way, every file included four generated texts based on the same book. Total cost was about 6,276,276 × .002 × 1.3 × .001 = US$ 16. We stopped text generation after this initially planned procedure was finished.

#### Word Frequency Estimation.

We used R for most of the data analysis and Python for text generation. We used the text mining package in R (tm; Feinerer et al., 2008) for measuring word frequency, using the default tokenizer, and removing punctuation and numbers using its “control” options. For lemmatization, we used UDPipe (Straka & Straková, 2017) with the default German treebank from the Universal Dependencies project (german-gsd; (R. McDonald et al., 2013). Similar to Table 2 in Schroeder et al. (Schroeder et al., 2015, we present an overview of the resulting corpus (see Table 1). Note that childLex used a different linguistic pipeline for tokenization and lemmatization (i.e., based on Geyken & Hanneforth, 2006; Jurish & Würzner, 2013), as we could not reproduce the original pipeline completely. Other than that, we tried to mimic the pipeline as well as possible.

**Table 1.** Size and descriptive statistics of the LLM corpus compared to childLex (Schroeder et al., 2015).

Measure	childLex	LLM corpus
n Books	500	500
Tokens	9,850,786	6,252,808
Types	182,454	46,409
Lemmas	117,952	34,519
% Hapax tokens	0.90	0.25
% Hapax types	48.74	33.03
% Hapax lemmas	48.30	33.09
% Tokens > 4	97.89	99.57
% Types > 4	26.53	41.81
% Lemmas > 4	27.91	41.24

As in childLex, we kept the original capitalization (sentences, nouns, etc.), since nouns in German are always capitalized. This makes our corpus more comparable and keeps as much structure in the corpus as possible. Note that this results in tokens such as *Essen* and *essen* (*food* and *to eat* in English) in the middle of sentences to be correctly counted as different types, but also tokens such as *Wahrscheinlich* and *wahrscheinlich* (*probably* in English) due to capitalization at the beginning of sentences. We used a log-transformed and normalized word frequency measure ($\log\left(\frac{\left(1+\text{frequency}\right)\times 10^{6}}{\text{corpus}\_\text{size}}\right)$), see Appendix Section “Word frequency transformation” for more details. For similar procedures and discussion, see Heister et al. (2011); Van Heuven et al. (2014).

#### Sources for Alternative Word Frequency Measures.

**Figure A.4.**
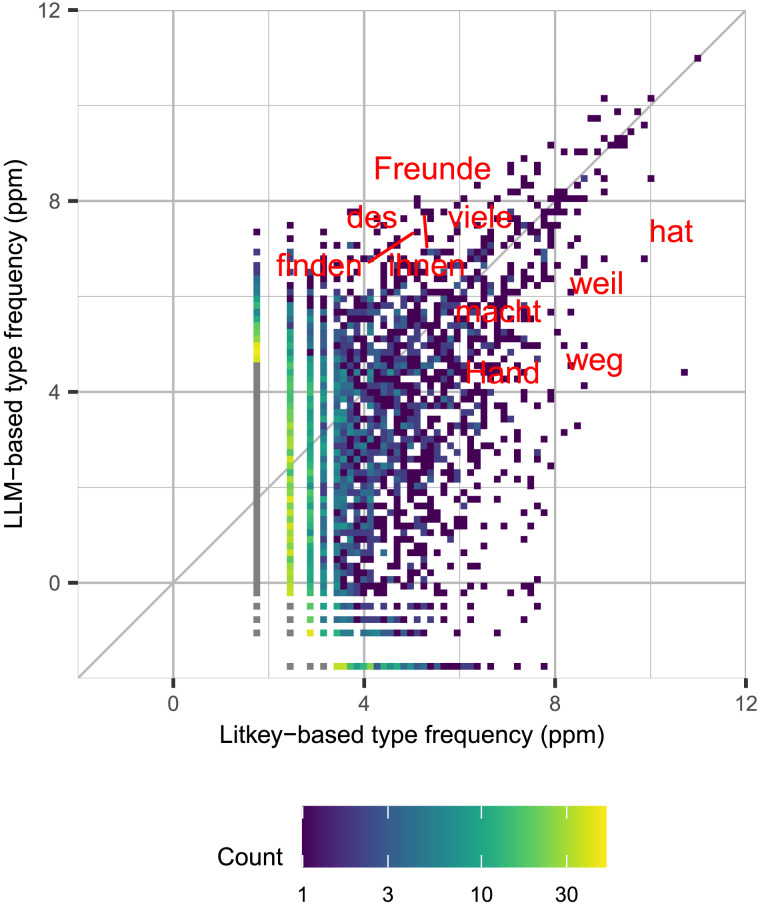
The same as Figure 3 in the main text, but showing Litkey-based type frequency (*x*-axis). The pattern is similar, despite much less available data.

We focus on the comparison with childLex (Schroeder et al., 2015). We also compare LLM word frequencies to word frequency measures from a number of other lexical databases. These include: Litkey (Laarmann-Quante et al., 2019), DWDS (Heister et al., 2011), SUBTLEX (Brysbaert et al., 2011), and Google Books (Brysbaert et al., 2016). Litkey is a considerably smaller corpus, but see the Appendix for a comparison with Litkey (Figure A.4).

#### Reading Performance Data.

We used the log-transformed lexical decision response times (RTs) on 1152 words as reading performance measures from DeveL (Schröter & Schroeder, 2017). The RTs represent the mean RT per word (*N* = 1152) and age group (Grade 1, 2, 3, 4, 6, younger and older adults). As is usually done, we applied a log transformation, which resulted in a more normal-like distribution of RTs and also improved model fit. Note that non-words were excluded, as the primary focus of the current analysis is the investigation of word frequency. Words in DeveL were selected in such a way that the low-frequency words (e.g., < 10 per million) accounted for only 25% of all words (in childLex, low-frequency words account for 95% of the words).

### Results

#### Word Frequency Distributions and Lexical Richness.

We compared several statistics between the LLM corpus and childLex (Schroeder et al., 2015). The generation procedure yielded an average of 625 words per generated text, totaling 6,276,276 words. Table 1 shows that this is about 66% of the size of childLex.

**Figure 1.**
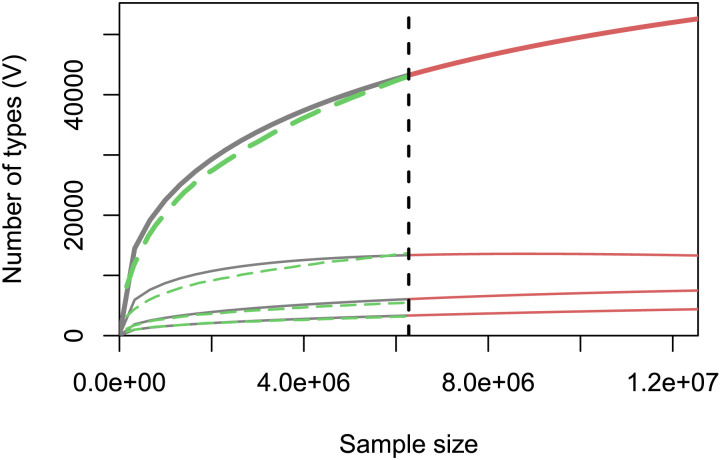
The type-token growth curves based on the LLM corpora show the increase in lexical richness as the sample size gets larger. The four curves show four different lexical richness measures (*y*-axis) on interpolated (solid grey), extrapolated (solid red), and observed (dashed green) sample size (*x*-axis), as based on a finite Zipf-Mandelbrot Large Numbers of Rare Events Model (LNRE model, see Evert, 2004). The four lexical richness measures are from top to bottom: total numbers of types, numbers of types that occur at most 3, 2, and 1 times (i.e., tris legomena *V*_3_, dis legomena *V*_2_, and hapax legomena *V*_1_).

To compare both corpora, we needed to normalize for sample size. After dividing by the total number of unique types and lemmas, we found a relatively low portion of unique types and lemmas in the LLM corpus (see % hapax types and lemmas in Table 1). We also evaluated the type-token pattern for different hypothetical sample sizes, see the topmost green dashed growth curve in Figure 1, which is similar to Figure 1 from Schroeder et al. (2015). The growth curve for childLex is more than twice as high, showing that the LLM produces lexically less rich language, i.e., with fewer unique types. In addition to observed numbers of types for subsets of the corpus, Figure 1 also shows predictions based on Large Numbers of Rare Events (LNRE) models (Baayen, 2001; Evert, 2004). These inter- and extrapolations show the expected change in lexical richness when corpus size increases. For both smaller and larger size corpora, lexical richness stays well below that of childLex. The percentage of hapax legomena (i.e., tokens occurring only once in the entire corpus) and unique types is much higher in childLex than in the LLM corpus. Words that recur at least five times (i.e., > 4; see Table 1) account for a larger portion in the LLM corpus. These findings provide a further indication that the LLM corpus is lexically less rich, which is in line with our subjective impression after reading the generated text.

**Figure 2.**
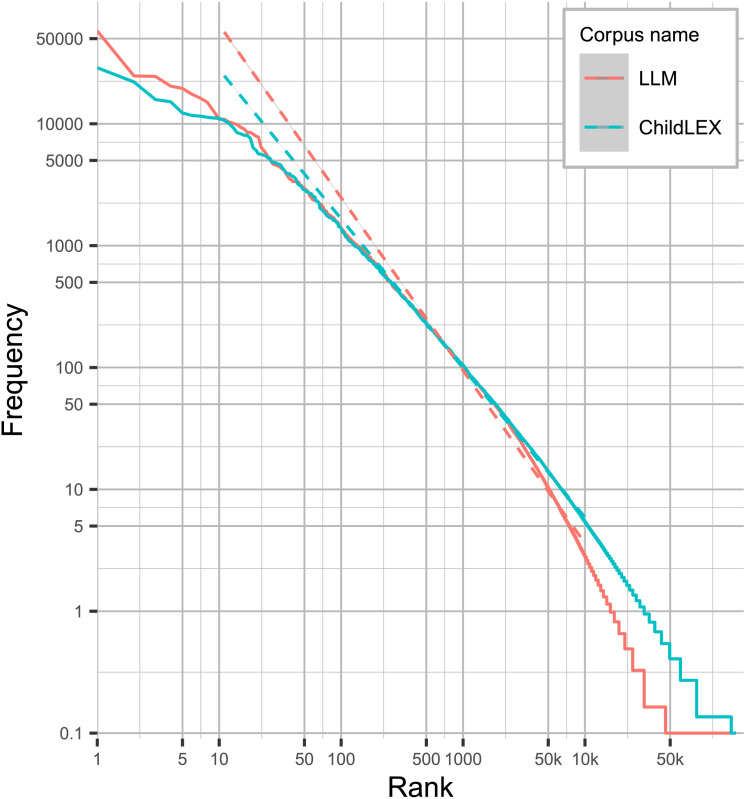
Zipf’s law plot shows frequency count on the *y*-axis and frequency rank on the *x*-axis with linear regression lines (dashed lines) for both the LLM corpus and childLex. The slopes are fitted to words with a frequency between 10 and 10,000. The LLM slope is more negative, indicating a higher occurrence of highly frequent words and a lower occurrence of low-frequency words.

More generally, we can also investigate this pattern by inspecting the balance of high and low-frequency words as indicated by Zipf’s law (i.e., word frequency is proportional to rank). This proportion in natural language corpora is never completely constant, so comparing the pattern across corpora can be interesting (see, e.g., Baayen, 2001, 2008; Piantadosi, 2014). Figure 2 shows that Zipf’s law in the LLM corpus is generally steeper compared to childLex. Previously, similar comparisons of adult-directed corpora showed the same pattern (i.e., SUBTLEX and Google Book corpus, Brysbaert et al., 2016). For the LLM corpus, the slope is steeper compared to childLex, indicating that the LLM corpus includes more high-frequency words and fewer low-frequency words. Again, this finding indicates that the LLM-based text is lexically less rich.

**Figure 3.**
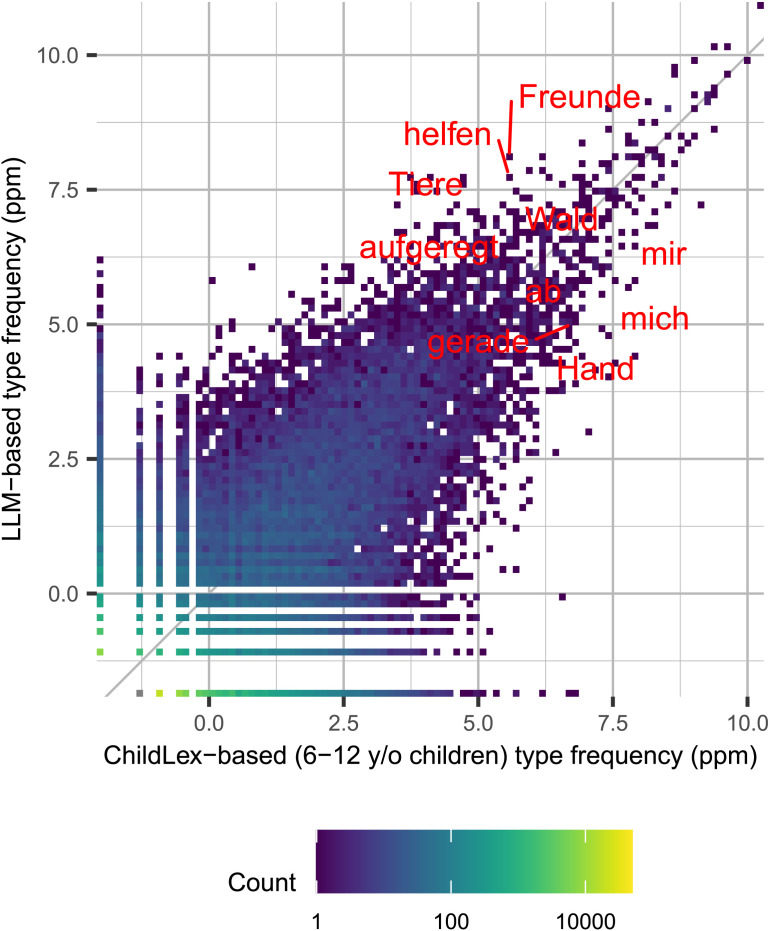
Correlation between LLM type frequency (*y*-axis) and childLex type frequency (*x*-axis; dark gray line). The labels show the top five differences on both sides (*x*-*y* and *y*-*x*). The color gradient of the dots represents the number of data points each dot represents.

Despite differences in lexical richness, we find a correlation between word frequency measures from childLex and the LLM corpus (*r* = .88 for frequency per million and *r* = .69 for log frequency per million, see Figure 3). The cone shape of the scatter plot (see Figure 3) indicates that lower frequency measures are noisier (more variable) than higher word frequency measures, which makes sense, given the lower numbers of observations for low-frequency words. The relatively symmetric shape indicates that this noise is similarly distributed between both corpora. The correlation remains .88 when we include only words that occur in both corpora. The correlation based on log frequency also stays the same. The high correlation and the scatter plot illustrate the high similarity of the two measures. Comparing word frequency in this way also allows us to look at the words that differ the most in both directions. In the LLM corpus, words that are more frequent sometimes result from spillover effects from the most likely predominant English training data. For example, the German word “namens” is used more often in the LLM corpus than in childLex (1414 vs. 31 per million). This could directly have “spilled over” from the typical phrase to start a story in English: “There was an X called Y” even though “namens” is not typically used in this context in German. This result is similar to the observed losses in lexical and morphological richness in automatic machine translation (Vanmassenhove et al., 2021). In contrast, word frequencies that stand out in childLex are typically associated with narrative storytelling. While this finding is unsurprising, childLex also contains some very common words that we would have expected to find more frequently in the LLM corpus. There is no clear explanation for these patterns, but some seem to indicate that childLex contains more colloquial style words (e.g., “offenbar”). Table 2 shows the most common words from both corpora that do not appear at all in the other corpus. In this table, the LLM column contains only names, which is a result of the way some common first names were removed from childLex but were kept in the LLM corpus. Unfortunately, we were not able to reconstruct the procedure used in childLex for removing given names. Table A.1 contains a more extensive analysis of such differences.

**Table 2.** The top frequent words that occur in only one of the two corpora, for all word lengths, and words with more than 10 characters. F represents the normalized log frequency. Some very common childLex words like “*offenbar*” (*evidently*), “*rasch*” (*quickly*), “*Hoffentlich*” (*Hopefully*), and “*guckte*” (*looked*) do not occur in the LLM corpus at all. Instead of “guckte” or “gucken”, words like “angeguckt” and “abgucken” do appear in the LLM corpus. Also, common first names have been removed from childLex, but not from the LLM corpus.

childLex	F	childLex > 10	F	LLM	F	LLM > 10	F
daβ	6.1	Hoffentlich	4.4	Max	8.4	nahegelegenen	4.3
	5.1	Brombeerkralle	3.8	Mia	7.1	Schulvampire	4.2
offenbar	5.0	Hosentasche	3.7	Tim	7.0	Tantenschreck	3.9
rasch	4.9	Augenbrauen	3.7	Lisa	7.0	SkaterBande	3.8
Eigentlich	4.7	Zeigefinger	3.5	Lena	6.7	ParkSheriffs	3.8
ehe	4.5	SternenClan	3.5	Anna	6.5	Lesefähigkeiten	3.8
Gleich	4.4	Olchi-Kinder	3.5	Emma	6.5	Schafgäääng	3.7
Hoffentlich	4.4	eingefallen	3.4	Tom	6.4	SchmuddelHund	3.7
glaub	4.4	kopfschüttelnd	3.1	Müller	6.2	Inselschüler	3.7
guckte	4.3	unwillkürlich	3.1	Lina	6.1	verwirklicht	3.6

#### Evaluating LLM Word Frequency Using Reading Performance.

We compared a linear regression model that included LLM word frequency measures based on the complete LLM corpus to linear regression models that included alternative word frequency measures. These other measures included childLex and two adult-directed corpora, a book-based corpus (DWDS Heister et al., 2011), and a subtitle-based corpus (SUBTLEX Brysbaert et al., 2011). We controlled for a number of other factors that affect reading performance, but that are not of interest to the current study: OLD20 (e.g., Hawelka et al., 2013; Yarkoni et al., 2008), age of acquisition (e.g., Weekes et al., 2006), word length (e.g., Gagl et al., 2015; Huestegge et al., 2009; Marinus & de Jong, 2010; Zoccolotti et al., 2005), as well as phoneme count, and uni-, bi- and trigram frequency. Note that for the final analysis, we removed the phoneme count and uni-, bi-, and trigram frequency parameters due to high Variance Inflation Factors (Fox & Monette, 1992). We removed all predictors with a Variance Inflation Factor below 5, which can be assumed to indicate low co-linearity (see Gregorová et al., 2023, for a similar procedure). After calculating the effect sizes from the linear model, we estimated the model fit based on the Akaike Information Criterion (AIC, Akaike, 1974). To estimate the AIC, we compared the model to a baseline model without a word frequency measure. Higher AIC differences indicate increased model fit.

**Figure 4.**
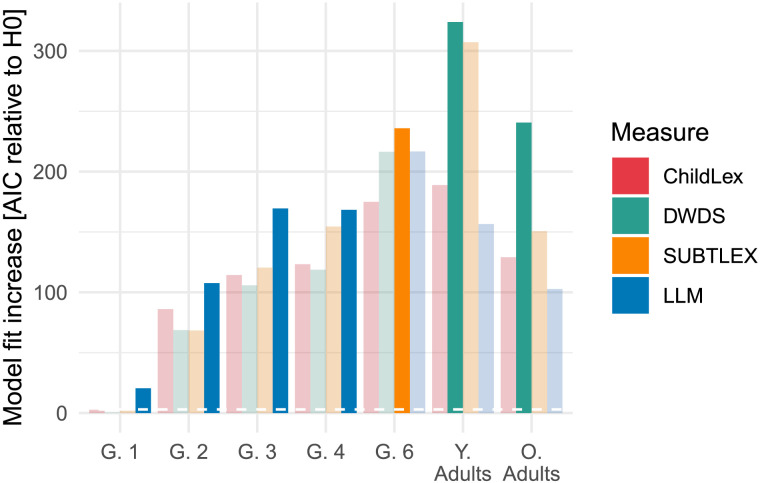
Evaluation of the word frequency effect on reading performance (RTs) for beginning readers (Grades 1, 2, 3, 4, and 6), younger and older adults. AIC difference is based on an analysis comparing the word frequency effect across LLM, childLex, DWDS, and SUBTLEX word frequency measures for all age groups. Models with the highest fit for each group are highlighted (i.e., the largest AIC difference). Note that G. stands for grade, Y. for younger adults, and O. for older adults. The white dashed line near the *x*-axis indicates the threshold for a significant model fit increase. Note that the age groups represent different data sets and that the bars are therefore only comparable within each age group.

We found that the AIC difference was largest for young readers (Grade 1–4; see Figure 4), indicating that LLM word frequency describes the word frequency effect in young readers best. For Grade 6, we found that SUBTLEX word frequency, which relied on subtitles from films and TV shows, had the highest model fit increase. Finally, book and newspaper-based word frequency from the DWDS corpus showed the highest model fit for younger and older adults, see Figure 4A. Table A.2 provides a description of model estimates, effect sizes, and the R-squared metric. When the word frequency increases from 1 to 1000, predicted RT decreases by about 250 ms. Appendix Section “Robustness analysis” shows a robustness analysis against sample size for the correlation with childLex word frequency as well as for the model fit on reading performance.

**Figure 5.**
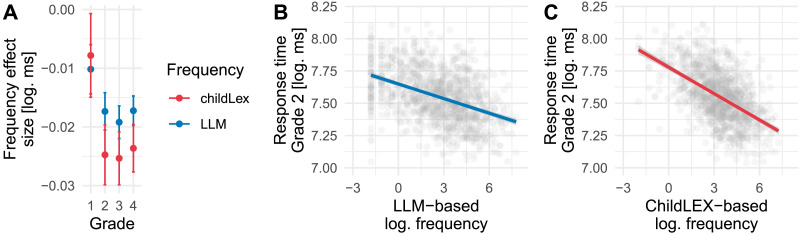
Evaluation of the word frequency effect size on reading performance (RTs) of beginning readers (Grades 1, 2, 3, and 4). (A) Effect sizes with 95% confidence intervals for log-transformed RT for the young readers of grades 1–4 for both LLM and childLex word frequency. (B) Scatter plot including linear regression line showing the word frequency effect on RTs for grade 2 readers based on the LLM and (C) childLex corpora.

The finding that LLM word frequency describes the word frequency effect in children best is accompanied by a reduction in effect size. When comparing the effect sizes of the word frequency effects between LLM and childLex word frequency, we found that for LLM word frequency, effect size tended to be smaller in Grades 2–4 (see Figure 5A). Inspecting the scatter plots of Grade 2 readers (as shown in Figure 5B and 5C and Table 3; even when OLD20 is removed) shows that the reduction in effect size is the combined result of the low-frequency measures and the words not included in the LLM corpus but do exist in childLex, indicating that these words play a crucial role for the adequate estimation of the effect size of the frequency effect in young readers.

**Table 3.** Comparison of the linear regression model results for Grade 2 readers using either the LLM-based or the childLex-based frequency measurement. Two models included the Orthographic Levenshtein Distance (OLD20) measure, which was based on childLex, and two models did not.

	*Grade 2 log. transformed reaction times*			
	LLM + OLD	childLex + OLD	LLM	childLex
log. frequency LLM	−0.017^***^		−0.018^***^	
	(0.002)		(0.002)	
log. frequency childLex		−0.025^***^		−0.025^***^
		(0.003)		(0.003)
Intercept	6.977^***^	7.045^***^	6.983^***^	7.052^***^
	(0.018)	(0.024)	(0.018)	(0.023)
OLD20	0.014*	0.011		
	(0.008)	(0.008)		
Age of Acquisition	0.047^***^	0.045^***^	0.048^***^	0.045^***^
	(0.003)	(0.003)	(0.003)	(0.003)
Letter length	0.062^***^	0.060^***^	0.064^***^	0.062^***^
	(0.003)	(0.003)	(0.002)	(0.002)
Observations	1,152	1,152	1,152	1,152
*R* ^2^	0.678	0.672	0.678	0.672
AIC	−1744	−1722	−1743	−1723

*Note:* *p < 0.1; **p < 0.05; ***p < 0.01.

### Summary: Experiment 1

LLM word frequency explained more variance in lexical decision response times compared to childLex word frequency, which is based on children’s books. We generated the text corpus based on 500 children’s book titles. In each prompt, we asked the model to compose a text for children in age-specific language, using the titles of the books contained in childLex (Schroeder et al., 2015). There were two main findings. (i) Word frequency correlated strongly between corpora, despite a lower lexical richness in the LLM corpus (based on the number of distinct words and words used only once). Frequent words comprised a more significant part of the LLM corpus compared to childLex. (ii) When evaluating response times, we found that LLM word frequency describes the performance of child readers better than childLex word frequency, indicating a lower effect size than previously assumed. In summary, this study demonstrates how an LLM corpus can represent word frequency and also reveals substantial differences compared to traditional corpora.

## EXPERIMENT 2

Experiment 2 investigates two of the findings from Experiment 1 in more detail. First, we investigate how increasing the temperature parameter influences the lexical richness of the corpus. Second, we investigate how prompting for child-directed vs adult-directed text influences model fit. To investigate the combination of these two factors, we generated four additional corpora: two corpora generated with child-directed and two generated with adult-directed prompts, of which one of each had the Experiment 1 temperature value and the other had an increased temperature value (i.e., 0.5 and 0.9). Together, these four corpora included: (i) *Child-Directed & Low Temperature (ChLT)*. (ii) *Child-Directed & High Temperature (ChHT)*. (iii) *Adult-directed & Low Temperature (AdLT*, as well as (iv) *Adult-directed & High Temperature (AdHT)*. Since ChLT used the same target audience and the same temperature, it is an attempt to reproduce the corpus from Experiment 1.

Like in Experiment 1, we compare the new LLM corpora to childLex (Schroeder et al., 2015) and fit the resulting measures to child reading performance. We also compare the fit for LLM word frequency with adult books-based word frequency (DWDS Heister et al., 2011) to determine which measure fits best to adult reading performance (DeveL contains response times for both children and adults). We expected higher model fits on child reading performance for child-directed word frequency compared to adult-directed word frequency. This would demonstrate that the LLM is indeed capable of generating text tailored to children. Likewise, we expected higher model fits on adult reading performance for adult-directed word frequency. For temperature, we expected that a higher temperature would increase lexical richness and, therefore, result in a corpus that is more similar to childLex. Given that in Experiment 1, the lexically richer corpus (childLex) resulted in lower model fits in reading performance, it can be expected that higher lexical richness would again result in a lower model fit to reading performance.

We decided to look at grades 1–4 together, as we assumed that reading across these grades is still in a comparable stage of development. Excluding adolescents follows developmental evidence that lexical processing shifts from reliance on frequency-based decoding (Grades 1–4, Age 6–9) to more automated recognition by Grade 6 (Meixner et al., 2022). Experiment 1 also showed differences between grade 6 and the younger children as well as the adult readers.

Compared to Experiment 1, we increased the size of both child-directed LLM corpora to ∼23 million words (going from ∼6 million in Experiment 1) to make sure that corpus size did not affect our findings. We expected this to increase the number of overlapping words with childLex and to increase the reliability of the low-frequency measures (Gernsbacher, 1984). This should also lead to more precise measures of lexical richness, which may be more important for a high-temperature setting.

### Methods

We used the same settings (“max_tokens” set to 4000) and prompt as in Experiment 1, except for the temperature (max. value was 2.0). Data was generated in June 2024 using GPT-3.5-turbo, which was now pointing to Snapshot Version 1106. In initial explorations, a temperature above 1.0 resulted in frequent hallucinations, such as changing the names of the main characters in the story (e.g., Ferdinand instead of Fred). A higher temperature also led to more incoherent stories. We decided to set the temperature to 0.9 in the high-temperature condition and maintain the same value as in Experiment 1: 0.5 in the low-temperature condition.

For each age group (Ages 1–5), we generated 10 texts for each of the 500 books in two runs, using both low- (0.5) and high-temperature (0.9) settings. Additionally, we generated one run of 10 child-directed texts per book without age specification for both temperature settings. In total, this resulted in 55,000 child-directed texts for each temperature setting. To generate adult-directed texts, we performed three runs of 10 texts per book, again using both temperature settings. This resulted in 15,000 texts for the low-temperature setting and 15,000 for the high-temperature setting.

We compared word frequency measures for both the child reading performance data (i.e., from Grades 1–4) and the adult reading performance data (i.e., younger and older adults).

### Results

#### Word Frequency Distributions and Lexical Richness.

The generated text was generally shorter compared to Experiment 1, averaging about 278 words per text for the high temperature conditions and about 270 for the low temperature conditions. Table 4 shows that all four crossed temperature and target audience conditions generally resulted in more unique types and lemmas compared to the LLM corpus from Experiment 1 (see Table 1). However, childLex was still much richer than all LLM corpora.

**Table 4.** Size and descriptive statistics for the 4 LLM corpora from Experiment 2 compared to childLex. Note that LT & HT refer to low and high temperature settings.

Measure	childLex	Adult LT	Adult HT	Child L	Child HT
n Books	500	500	500	500	500
Tokens	9,850,786	7,191,531	7,368,921	23,320,466	23,887,118
Types	182,454	71,423	83,921	84,978	110,603
Lemmas	117,952	52,528	61,318	63,552	82,126
% Hapax tokens	0.90	0.34	0.44	0.12	0.18
% Hapax types	48.74	34.69	38.21	33.56	38.21
% Hapax lemmas	48.30	34.82	38.13	33.00	37.05
% Tokens > 4	97.89	99.41	99.29	99.79	99.71
% Types > 4	26.53	40.51	37.26	42.48	37.38
% Lemmas > 4	27.91	40.26	37.13	42.82	38.10

For the adult-directed LLM corpora, the higher number of unique types, compared to the LLM corpus from Experiment 1, is plausibly due to the adult-directed audience prompt change creating a richer vocabulary (i.e., see ∼46k in Experiment 1 vs. ∼71k and ∼84k in the adult-directed LLM corpora). For the child-directed corpora, it is likely that the increase in unique types is due to a larger corpus size (i.e., see ∼46k in Experiment 1 vs. ∼85k and ∼111k in the child-directed corpora from Experiment 2). For the child-directed corpora, the % of hapax tokens (i.e., % hapax legomena across all tokens, meaning the percentage of tokens in the corpus that occur only once) was lower compared to Experiment 1 (.12% and .18% vs. .25%). This is also plausibly due to a larger corpus size, as the number of hapax legomena does not increase substantially for larger sample sizes (see the differences in slopes of Figures 1 and B.4. The % of hapax legomena across all types or all unique lemmas remained relatively constant with a larger corpus size (Types: Exp. 2: 33.56% vs. Exp. 1: 33.03%; Lemmas: Exp. 2: 33.00% vs. Exp. 1: 33.09%).

Furthermore, the high temperature setting has a stronger influence on the percentage of hapax types than having an adult target audience. In other words, the temperature setting more strongly influenced the percentage of words used only once, compared to the target audience. This can be illustrated by comparing percentages: 38.21% for ChHT vs. 33.56% for ChLT is a larger difference than 34.69% for AdLT vs. 33.56% for the ChLT.

Taken together, generating texts with a high temperature setting and prompting for adult text increased the lexical richness. However, lexical richness was still substantially lower than the lexical richness in childLex (48.74% hapax types in childLex vs. < 38.21% in the LLM corpora).

Using the growth curves from extrapolated sample sizes, we also see that the lexical richness in both adult-directed corpora was higher than in the child-directed corpora (see Figure B.4). The number of types, at around 20 million tokens (2 × 10^7^), is around 90,000 types for the AdLT (adult-directed low temperature, see above) corpus and 75,000 types for the ChLT corpus. Likewise, there are about 105,000 types for the AdHT corpus and only 95,000 types for the ChHT corpus (i.e., roughly an increase of 10,000–15,000 types for the adult-directed corpora). These differences are smaller compared to the temperature manipulation (90,000 vs. 105,000 types in adult-directed corpora and 75,000 vs. 95,000 types in child-directed corpora). At 10 million tokens, childLex already has more than double the number of types (about 80,000 types for AdLT, 90,000 types for AdHT, 60,000 types for ChLT, and 70,000 types for ChHT).

**Figure B.1.**
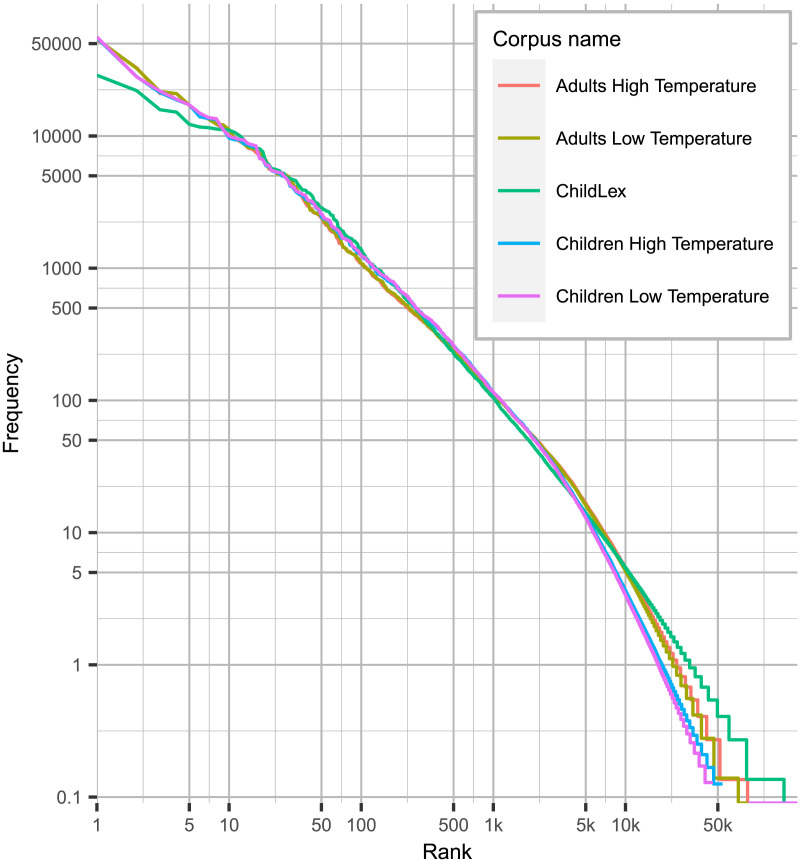
Zipf’s law plot showing a stronger negative slope for the LLM corpus compared to childLex. The slopes are fitted to words with a word frequency between 10 and 10,000.

This pattern is also confirmed by comparing the relation between word frequency and rank for the three new conditions, as in Figure 2 from Experiment 1 (see Figure B.1). The three new conditions fall between the curves from Experiment 1 and the curve based on childLex. The AdHT curve matches childLex most closely.

**Figure 6.**
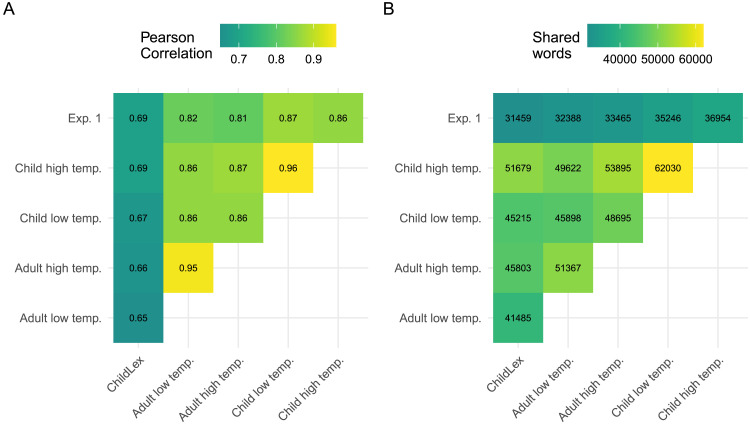
(A) Pairwise correlations between log word frequency measures derived from the LLM corpora and childLex. (B) Number of shared words between corpora.

The correlation between LLM and childLex log word frequency was slightly smaller compared to the correlation we found in Experiment 1 (*r* = .69 vs. .67, see Figure 6A). Word frequency from Experiment 1 correlated most strongly with those from the reproduction condition (ChLT; *r* = .87). Figure 6B shows the varying numbers of shared words on which the pairwise computed correlations are based. We find higher correlations for the corpora with the same vs. different target audience, regardless of temperature (see Figure 6A). In contrast, as we showed above, the percentage of hapax words across types or lemmas is more similar within vs. between temperature settings, illustrating the higher lexical richness due to increased temperature.

The percentages of shared words exhibit the same pattern observed in the correlations (see Figure B.3): the same target audience yields more overlapping words compared to using the same temperature setting. Also, the ChHT corpus contained the highest percentage of words from childLex (33%, see the first column for the ChHT row in Figure B.3). This suggests that child-directed prompts result in a better overlap with childLex than adult-directed prompts, and also that a higher temperature results in more recovered words. Also, compared to the 20% shared words of Experiment 1, this marks a notable increase. Vice versa, childLex contained the most words from the AdLT corpus (58%, see second column for the childLex row), probably because this corpus was the smallest with the least types. The overlap between the LLM corpora ranged from 33% to 73%. This suggests that corpus size, temperature, and target audience yield relatively distinct statistics.

The words in the LLM corpus that do not occur in childLex are very similar to those from Experiment 1 for both the low- and high-temperature conditions. ChildLex words that do not occur in the LLM corpus are now different, which is plausible, given the larger corpus from Experiment 2. The words missing from the LLM corpus seem heterogeneous, including words such as *immerhin* (anyway), see Tables B.3 and B.7, or *mich* (mine), see Tables B.4 and B.8. Even though they are missing here, it is not implausible that such words will eventually occur as an LLM corpus gets larger, given their large amounts of training material.

#### Evaluating LLM Word Frequency Using on Reading Performance.

**Figure 7.**
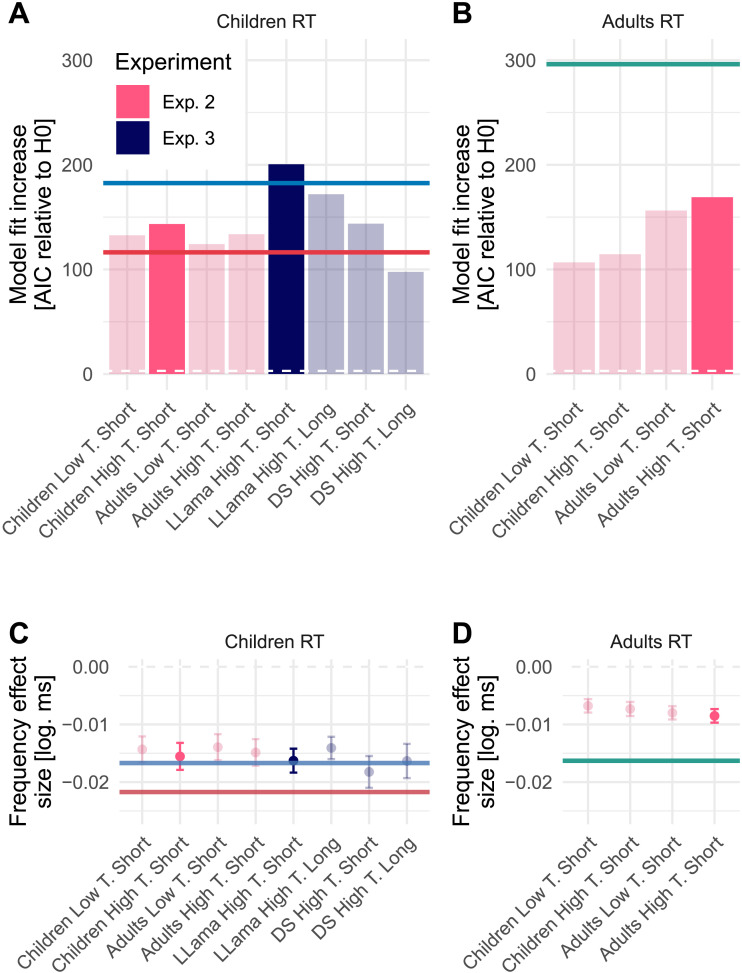
Model comparison and effect size results for children (A and C) and adults (B and D). A and B show AIC differences based on linear mixed-effect regression models of RTs with and without the word frequency measure. C and D show corresponding word frequency effect size estimates, including 95% CIs. The solid blue and red lines in panels A and C represent Experiment 1 LLM word frequency and childLex, respectively, and the solid green lines in panels B and D represent DWDS (which was the best-performing model for the adult RTs in Experiment 1). Note that Experiment 2 results are color-coded in red and Experiment 3 results are color-coded in dark blue. The best-fitting models in each Experiment are highlighted by more intense colors.

As in Experiment 1, we estimated model fit increase (i.e., AIC) in relation to a baseline model (i.e., without word frequency included). Again, all LLM-based models resulted in a larger increase in model fit compared to the childLex-based model when fitted to the child RTs (see Figure 7A). However, the Experiment 1 LLM corpus, even when generated with a low-temperature setting, still had the highest model fit. For the adult RTs, word frequency based on adult-directed text resulted in a higher model fit compared to child-directed text. DWDS word frequency resulted in the best fit (see Figure 7B).

All effect size estimations had confidence intervals that excluded 0, indicating a significant word frequency effect. The CIs also all excluded the estimated effect size for childLex, indicating that LLM word frequency results in weaker estimations of the word frequency effect (see Figure 7C and D).

We found that age-specific corpus generation enables a more accurate estimation of the word frequency effect on child reading performance of children compared to adults. We compared the child-directed LLM word frequency fitted to both adult and child reading performance data and vice versa (see Figure 7A vs. B). We did not observe differences in estimated effect sizes (overlapping CIs).

Surprisingly, an increase in temperature results in better model fit for both the child- and adult-directed word frequency measures and also for both the child (see Figure 7A) and adult RTs (see Figure 7B). The estimated effect sizes remain similar to the Experiment 1 estimate (see the overlapping CIs in Figure 7C and D).

### Summary: Experiment 2

Experiment 2 replicated the main finding of Experiment 1: a word frequency measure derived from LLM corpora can be used to model the word frequency effect in reading effectively. Experiment 2 manipulated two more variables: 1) the LLM temperature parameter (high vs. low) to try to increase the lexical richness of the corpus, and 2) the target audience (adults vs. children) to investigate if prompting for child-directed text makes a difference.

We found that a higher temperature increased the lexical richness of the corpora and that higher temperatures resulted in higher model fits. Confirming our assumption for Experiment 1, we found evidence that LLM corpora can indeed be tailored to a specific target audience. However, the finding that model fit increases with increased lexical richness is a new facet not predicted by the findings of the first study. Overall, LLM word frequency from Experiment 1, with a low lexical richness, showed the highest model fit for children. Notably, the corpus with the same settings as in Experiment 1 (i.e., child-directed and low temperature) also resulted in a lower model fit. This finding suggests that using the same model but different snapshots can yield significantly different results. The main issue is that the LLM that we used for Experiments 1 and 2 is closed-source, motivating the use of an open-source model in Experiment 3, which potentially allows for a more reproducible use of LLMs. For adults, model fit increased with lexical richness, but could not reach the model fit for the book-based DWDS word frequency.

## EXPERIMENT 3

Experiment 3 had three goals: (i) We aimed to replicate the Experiment 1 results using two different open-weight LLMs, making sure that our findings are computationally reproducible across models. (ii) We aimed to explore the effects of increasing the length of generated text. We expected an increase in lexical richness for corpora containing longer texts. (iii) In Experiment 1, we found a stronger model fit to child reaction times for LLM word frequency compared to childLex word frequency, despite having lower lexical richness. This indicated that a higher lexical richness does not necessarily lead to better model fit. For Experiment 3, we aimed to further investigate whether higher lexical richness again results in lower model fit to child reaction times (RTs). We adopted a similar procedure to that in Experiment 2, generating four corpora from two different LLMs with two different text lengths, thereby creating another 2 × 2 design.

### Methods

#### Model Choice and Prompt.

We determined that suitable models must have the capability to generate text in German, have at least open-source weights (ideally also code and full documentation), and not be a distillation, combination, or foundation model. We determined currently available and high-performing LLMs using Chatbot Arena (Chiang et al., 2024) and the Hugging Face open LLM leaderboard (Fourrier et al., 2024) in February 2025. Based on these considerations, we selected Llama-3.3-70B-Instruct (Grattafiori et al., 2024) and DeepSeek-V3 (A. Liu et al., 2024) to have models with different architectures and numbers of parameters. DeepSeek is a Mixture-of-Experts (MoE) model with 671B total parameters, with 37B activated for each token. Llama 3.3 70B is a dense transformer model with 70B total parameters, all of which are typically active. Weights and limited code and documentation are available, but no training data or peer-reviewed papers are available, among other issues (see https://osai-index.eu for an evaluation).

We generated short-text corpora with a prompt similar to Experiments 1 and 2: *“Schreibe eine Kindergeschichte für Kinder im Alter {age} über: {book_title}”* (write a children’s story for children aged {age} about {book_title}). This small prompt change compared to Experiments 1 and 2 ensured generation of stories rather than commentary in the two LLMs used here. For the long-text corpora, we experimented with similarly simple but also more complex prompts (e.g., first prompt: describe the first quarter of the book; second prompt: describe the second quarter of the book; etc.). We found that a continuation-based strategy worked best: The first prompt was always the same as for the short-text corpora, and all follow-up prompts were: “Continue from: ” + currenttext[−500:], until reaching 4000 words. This prompted the model with the last 500 characters of the current output to improve topical consistency and reduce the generation of generic text.

#### Procedure.

For the short-text corpora, we conducted 50 runs of text generation for each of the 500 book titles. To reach a text length similar to Experiment 2, we set the max_tokens parameter to 600 for Llama and 700 for DeepSeek, after experimenting with different settings. Text generation resulted in an average of 287 (Llama) and 284 (DeepSeek) tokens per text, with a total corpus size of about 8 million. For the long-text corpora, we conducted five runs of text generation for each of the 500 book titles. We set max_tokens to 2000, and we configured the prompt to continue producing text until at least 4000 words per book were generated, which resulted in an average of 4155 (Llama) and 4031 (DeepSeek) words per text. Following our findings from Experiment 2, we set the temperature parameter to 0.7 for all generated texts. We also retained the default settings of other parameters in both models (top_p = 0.7 and top_k = 50). For the long corpora, the total corpus size was similar to that of childLex (see Table 5). We used meta-llama/Llama-3.3-70B-Instruct-Turbo and deepseek-ai/DeepSeek-V3 (not the updated DeepSeek-V3-0324, which was not yet available) deployed on Together.ai (https://api.together.ai/models) in March 2025. Note that “Turbo” only indicates that the model is quantized to FP8, a numerical format used for deployment at large scale with minimal impact on model output. The cost was US$ 0.88 per million tokens for Llama and US$ 1.25 per million for DeepSeek. The DeepSeek model resulted in frequent connection errors and significantly slower text generation speed than Llama.

**Table 5.** Size and descriptive statistics for the four corpora from Experiment 3 compared to childLex. Llama is Llama-3.3-70B-Instruct, and DS-V3 is DeepSeek-V3.

Measure	childLex	Llama long	Llama short	DS-V3 long	DS-V3 short
n Books	500	500	500	500	500
Tokens	9,850,786	10,332,850	7,215,565	9,763,062	7,162,685
Types	182,454	51,320	40,660	239,830	95,321
Lemmas	117,952	39,272	39,272	191,309	74,695
% Hapax tokens	0.90	0.17	0.19	1.43	0.62
% Hapax types	48.74	33.62	34.01	58.10	46.52
% Hapax lemmas	48.30	34.13	34.13	55.39	44.60
% Tokens > 4	97.89	99.71	99.67	98.03	99.06
% Types > 4	26.53	42.19	42.09	20.00	29.54
% Lemmas > 4	27.91	41.49	41.49	21.53	30.46

We extended the pre-processing pipeline from Experiments 1 and 2 by applying additional normalization to the frequency list. After removing numbers/punctuation, we further cleaned the data by removing remaining special punctuation, non-alphabetic characters (except German diacritics), and consolidating whitespace. Duplicate word forms were merged under their normalized versions while retaining original forms for reference. This reduced noise from orthographic variants while preserving linguistic content. The additional normalization had only a marginal effect on the results.

### Results

#### Word Frequency Distributions and Lexical Richness.

**Figure 8.**
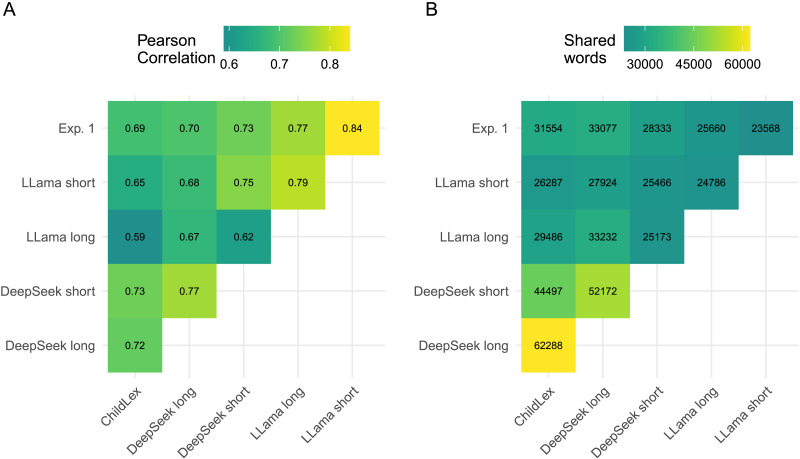
(A) Pairwise correlations between log word frequency measures derived from the DeepSeek and Llama corpora. For comparison, we additionally included the LLM corpus from Experiment 1 and childLex. (B) Number of shared words between corpora. Numbers and color scale represent Pearson correlations in A and the number of shared words in B.

**Figure C.1.**
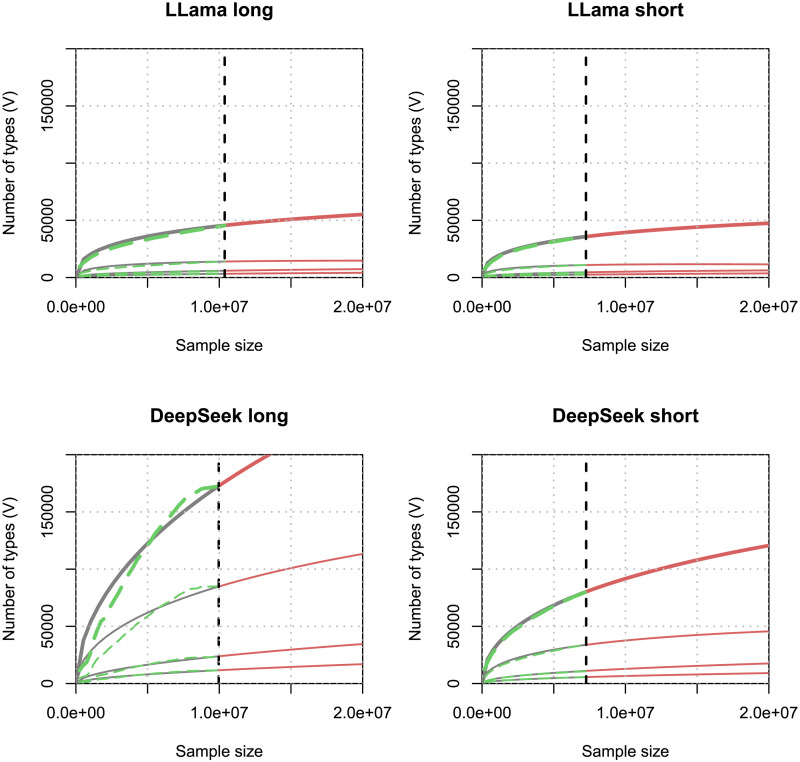
Similar to Figures 1 and B.4, these type-token growth curves show the dependency of the total number of unique types (*y*-axis) on inter- and extrapolated sample sizes (*x*-axis) for both Llama (top), and DeepSeek corpora (bottom).

**Figure C.2.**
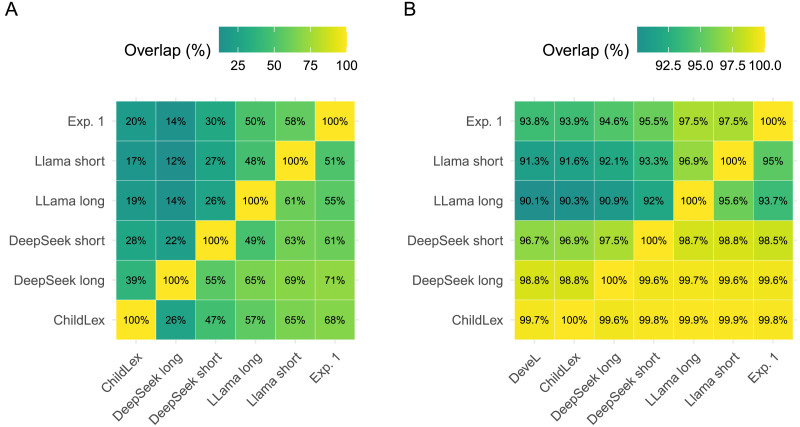
Overlap measured as percentage of retrieved words from one of both corpora. Overlap was defined as a word frequency > 0 for both corpora. The overlap percentages were computed by taking the number of words shared between the corresponding corpus listed on the left and bottom and dividing that number by the total corpus size of the corpus listed on the bottom row. Panel A shows percentages for complete corpora while panel B shows overlap for the DeveL selection of words only.

Lexical richness was higher in both DeepSeek corpora compared to both Llama corpora and all Experiment 1 and 2 corpora, see % hapax tokens and types in Table 5. The short-text DeepSeek corpus had a slightly lower lexical richness and the long-text corpus had a higher lexical richness than childLex (e.g., .62% and 1.43% words occur only once vs. .90% for childLex; see Table 5). Figure C.1 also shows growth curves of these values for different corpus sizes. The word frequency correlations are also higher for both DeepSeek corpora, and the number of shared words is highest for the DeepSeek long-text corpus (see Figure 8). Normalizing for corpus size, the long-text DeepSeek corpus uses about a third of the childLex words (39%, see Figure C.2). In contrast, the Llama corpus included only about one-sixth of the childLex words (this was similar to the Experiment 1 overlap and lower than the Experiment 2 overlap, see Figure B.3). The long-text DeepSeek corpus generated the highest number of types overall, resulting in an increase of about 50 thousand types compared to childLex (i.e., see Table 5). Thus, the long-text DeepSeek corpus contained both many childLex words as well as words not in childLex (see Tables C.1 and C.2 for examples).

For the Llama corpora, we found similar lexical richness to that of the GPT-based corpora, again showing lower lexical richness compared to childLex (i.e., see Table 5). We found no clear difference between long and short Llama corpora. Llama resulted in lower word frequency correlations with childLex and had lower overlap with childLex compared to DeepSeek (see Figure C.2).

#### Evaluating LLM Word Frequency Using Reading Performance Data.

**Figure 9.**
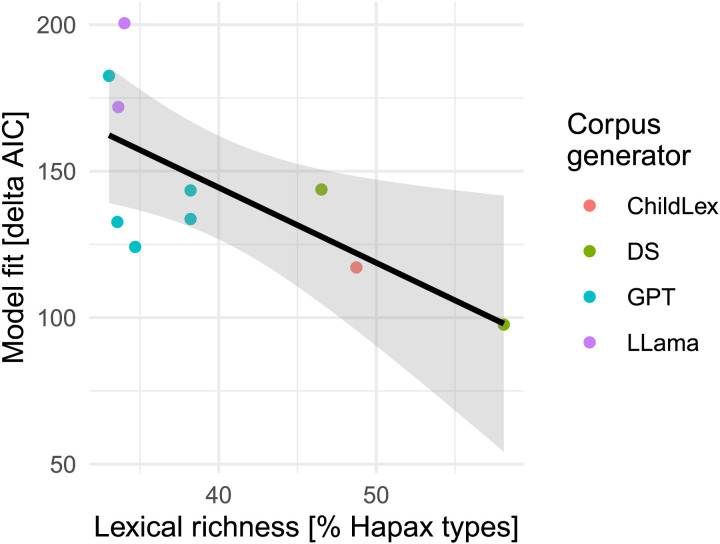
Model fit over lexical richness (measured by % hapax tokens) across nine LLM generated corpora and childLex.

We found that word frequency based on the Llama short-text corpus resulted in a higher model fit to child RTs compared to all other word frequency measures generated across Experiments 1, 2, and 3 (see Figure 7A). In contrast, both DeepSeek corpora performed less well. The word frequency measure based on the lexically rich long-text DeepSeek corpus was the only LLM-based measure that resulted in a lower model fit compared to the childLex word frequency measure. Still, all effect size estimates were highly comparable (see Figure 7B). Together, these results are in line with the observation that corpora with higher lexical richness are less well-suited to explain reading performance in young readers. Indeed, when we compiled all model fits and lexical richness estimates from the LLM corpora and childLex generated across all three experiments, we found a strong correlation (*r* = −.69, see Figure 9).

### Summary: Experiment 3

In Experiment 3, we extended our study using two open-weight LLMs (Llama-3.3-70B-Instruct and DeepSeek-V3), generating short- and long-text corpora. DeepSeek generated text with similar or higher lexical richness than childLex, although both LLMs generated unusual word combinations rarely used in natural language (e.g., see Table C.1). The Llama-based corpora were less lexically rich, but the extracted word frequency measures described the word frequency effect in reading performance better than any other corpus; in particular, for the short-text Llama corpus. Combining the results from all three experiments thus showed that corpora with lower lexical richness generally resulted in better model fits. Experiment 3 did not further investigate the role of the temperature parameter.

## GENERAL DISCUSSION

In three experiments, we explored whether word frequency derived from LLM text is adequate for estimating the word frequency effect in visual word recognition. We decided to focus on German children because we expected to need a smaller corpus, and relevant resources are available in German.

In Experiment 1, we generated an LLM corpus based on child-directed texts using GPT-3.5-turbo. The corpus was less lexically rich compared to a corpus of child-directed texts written by humans (childLex; Schroeder et al., 2015). Compared to childLex word frequency, LLM word frequency better explained variability in child reading performance (lexical decision reaction times for 1000+ words taken from DeveL; Schröter & Schroeder, 2017). Interestingly, when comparing the size of the word frequency effect, the better-fitting LLM word frequency resulted in a lower effect size.

Experiment 2 demonstrated that using child-directed prompts indeed results in a word frequency measure that is adjusted to child RTs. When we generated text with child-directed prompts, it was less lexically rich and fitted better to child RTs. When we generated text with adult-directed prompts, it was more lexically rich and fitted better to adult RTs. Experiment 2 also showed that increasing the temperature can increase lexical richness, but not to the same level as that of childLex. All four Experiment 2 corpora, including the reproduction condition, resulted in lower model fit to child RTs compared to the Experiment 1 corpus. This was likely a result of using a newer snapshot LLM version in Experiment 2, as this was the only difference from the Experiment 1 generation procedure.

In Experiment 3, we generated short- and long-text corpora using open-weight LLMs. Llama generated text with relatively low lexical richness, while Llama word frequency described lexical decision times best (i.e., model fit was better compared to all other LLM-based frequency measures). DeepSeek generated corpora with a higher lexical richness than every other corpus, while DeepSeek word frequency did not result in a good model fit. In a final analysis, we correlated the lexical richness and model fit of all available corpora and demonstrated that the model fits decrease with increasing lexical richness of a corpus. In summary, we generated LLM corpora with varying target audiences, temperature settings, closed- and open-weight LLMs, and text lengths. LLM word frequency captured the word frequency effect in child reading across all corpora, and effect size estimations were similar.

### Lexical Richness of LLM Corpora

**Figure C.3.**
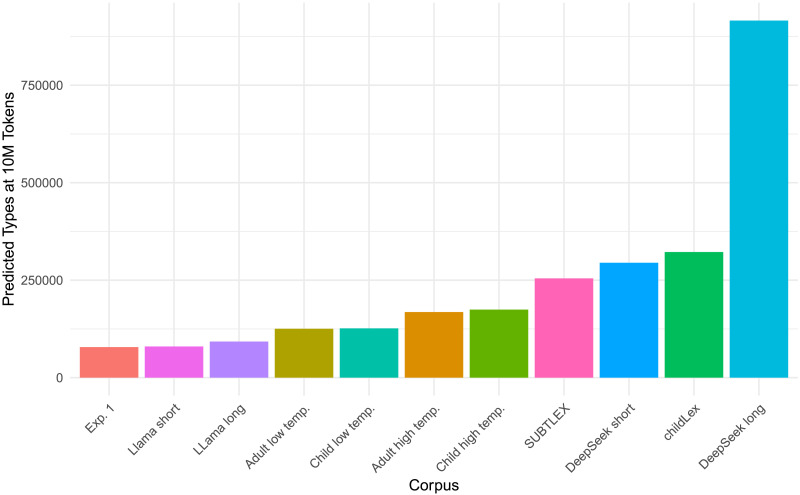
Based on the estimated growth curves in Figures 1, B.4, and C.1, we determined the predicted number of types for a hyphotetical 10-million-token corpus. The order of types turned out to be the same as the ordering based on the percentage of hapax tokens.

Corpus comparison revealed that all but one LLM corpus contained fewer unique types than the childLex corpus. Measures of lexical richness included the percentages of hapax legomena, see Figure 9, and the predicted number of types based on LNRE models, see Figure C.3. In line with this, word frequency distributions generally also include more frequent types. We could increase lexical richness by (i) increasing the temperature, (ii) prompting for adult-directed text, (iii) increasing the text length, and (iv) using different LLMs. We observed a comparably large increase in lexical richness for DeepSeek but not for Llama, especially for long text lengths. This finding could be attributed to the larger number of model parameters, training data, or the architecture used, among other factors. Explaining this pattern in detail requires additional analysis, as well as greater transparency in, for example, training data (Liesenfeld et al., 2023).

What does lexical richness of LLM text indicate? The results of Experiment 2 rule out that low lexical richness is simply the result of using child-directed prompts or a low temperature value. Extrapolation also showed that if the LLM corpora would have had the same size as childLex (10 million tokens), they would have had roughly less than half of the types (300k types for childLex vs. somehwere between 75k and 175k types, depending on the corpus), see Figure C.3. The lexical richness of childLex is roughly comparable to SUBTLEX and other existing adult-directed corpora (Baayen, 2001).

It has been observed before that LLM performance can suffer when confronted with words that do not appear often in training data across a number of cognitive tasks, including reasoning Razeghi et al. (2022) and answering fact-based questions Kandpal et al. (2023). If such biases exist in low-frequency words, it seems reasonable for LLMs to show a preference for higher-frequency words when prompted to generate text. The low-frequency words that the LLMs used in our study were often unusual combinations or derivations of other words (e.g., *Vollmond-tanz*, meaning “full-moon-dance” and *Lieblings-Sonnbrille*, which translates to *favourite-sunglasses*). Tables C.1 and C.1 include some of the words not in childLex or that occur the least often in childLex. In the DeepSeek-long corpus, this resulted in higher lexical richness, but the resulting set of DeepSeek words was still considerably different from childLex. For example, out of the 180k words in childLex, the DeepSeek long-text corpus included only about 60k words (i.e., only about 1/3 of the childLex). Still, this overlap was the highest across all LLM corpora. Thus, the high lexical richness of the DeepSeek long-text corpus results from 60k words that overlap with childLex and about 180k words that do not overlap with childLex.

Despite the low richness of much of the LLM text that we generated, LLM text still resulted in word frequency measures that are similar to existing word frequency measures. We observed a moderately strong correlation between childLex and the Experiment 1 LLM word frequency (.69 for log-frequency per million across ˜50,000 words), which seems typical compared to previously reported correlations. For example, SUBTLEX-UK (an English subtitle-based corpus) correlated at *r* = .66 to the Children Printed Word Database (˜9,000-word frequencies across 11,000 English children books, (Van Heuven et al., 2014) using a different but similar transformation of the word frequency counts. The LLM text thus does seem to represent certain qualities of natural text.

It was clear that LLM text remained qualitatively different from human-written text. LLM-based text was more stereotypical, while narrative, figurative, and rhetorical elements were often missing or felt unnatural. Subjectively, different LLMs yielded distinct styles. LLMs more often generated words central to the prompted book (e.g., *wand* in Harry Potter) and used fewer function words and word types that indicate direct speech, such as “sagt” (*says*). Future efforts could focus on prompting for book-length text (i.e., as shown here or here) to increase lexical richness and improve coherence etc. The corpus containing longer texts generated with DeepSeek did show a comparably high lexical richness, but this was also the corpus that subjectively contained the least coherent stories. Although we used lexical richness as a measure to characterize related differences in comparison with childLex, it is necessary to perform more detailed comparisons with other corpora as well as study other lexical, grammatical, and semantic statistics, including entropy, complexity, word frequency profiles, embeddings etc. (Dentella et al., 2023; Hu et al., 2024; Muñoz-Ortiz et al., 2024; Wu et al., 2024). Kumarage and Liu (2023) applied stylometric analyses to LLM texts and found that GPT 3.5 and 4 styles do not differ substantially from each other, relative to open-source LLMs. More research is necessary to determine the literary capabilities of modern LLMs compared to humans. Based on our findings, the relatively low lexical richness of LLMs could be troubling for using them to study child lexicon development (Korochkina & Rastle, 2025) and educational uses in general (see also Kasneci et al., 2023).

### Evaluation Based on the Word Frequency Effect in Reading Performance.

We estimated the effect of word frequency in child reading performance to test whether LLM word frequency could characterize their lexical memory or how they access their mental lexicon (Brysbaert et al., 2011, 2018). LLM word frequency allowed an adequate estimation of the word frequency effect while controlling for relevant covariates (age of acquisition, OLD20, word length). Most word frequency measures, except for the measure based on the DeepSeek short-text corpus, resulted in higher model fit than the children’s book-based frequency measure. Word frequency from less rich LLM corpora best fits the data, with the Llama short text corpus showing the highest model fit.

**Figure B.2.**
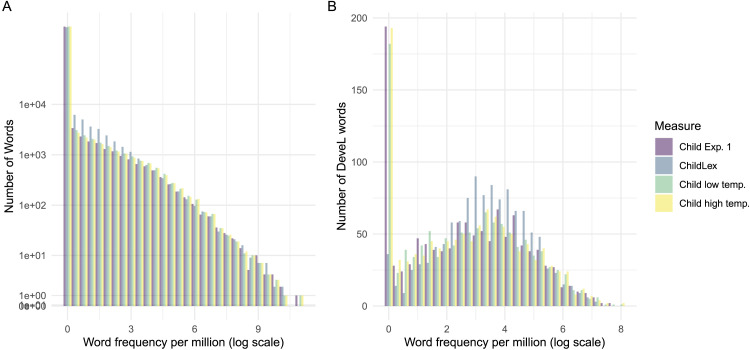
Distributions of word frequency based on childLex and three LLM corpora for all words (left) DeveL words only (right). For clarity, we grouped values with a log frequency per million that was lower than 0.

**Figure B.3.**
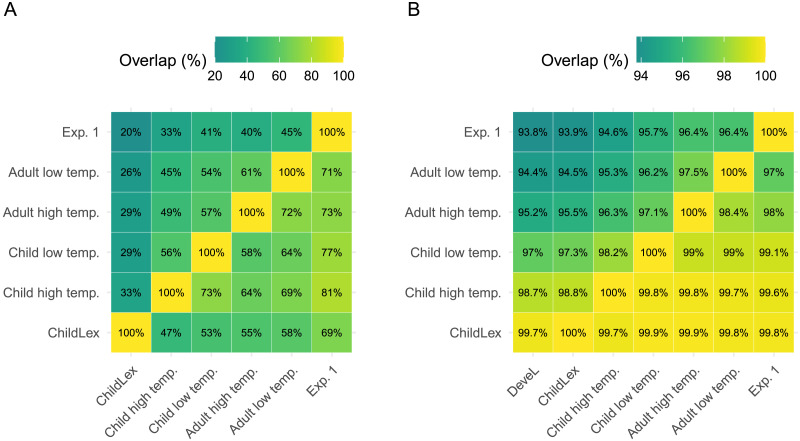
Overlap measured as percentage of retrieved words from one of both corpora. Overlap was defined as a word frequency > 0 for both corpora. The overlap percentages were computed by taking the number of words shared between the corresponding corpus listed on the left and bottom and dividing that number by the total corpus size of the corpus listed on the bottom row. Panel A shows percentages for complete corpora while panel B shows overlap for the DeveL selection of words only.

**Figure B.4.**
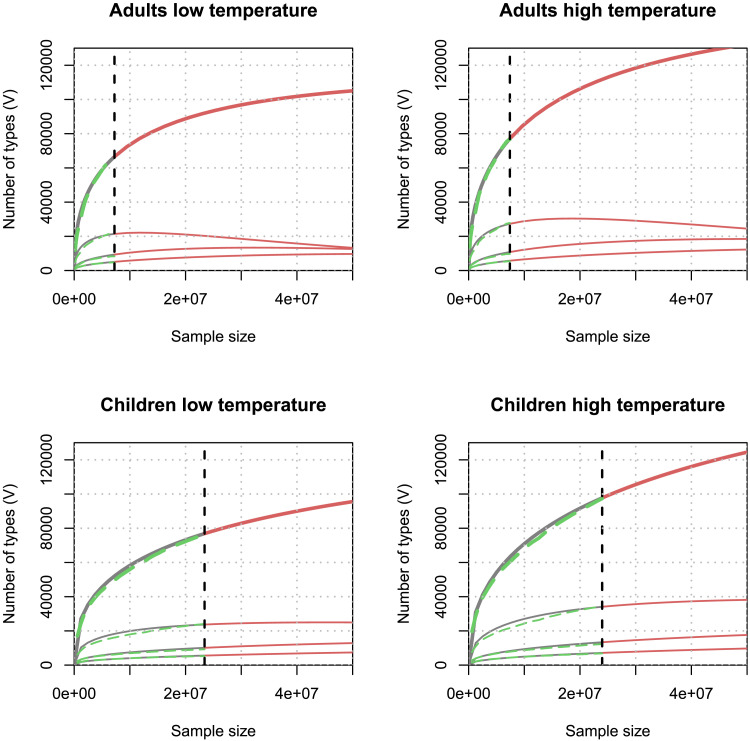
Similar to Figure 1 (the type-token growth curve from the main text), these type-token growth curves show the dependency of the total number of unique types (*y*-axis) on inter- and extrapolated sample sizes (*x*-axis) for both adult-directed corpora (top), and for both child-directed corpora (bottom).

The estimated size of the frequency effect was lower than we expected. Except for grade 1, childLex word frequency showed a larger effect size. Also, for adults, the frequency estimate that resulted in the best-fitting model showed a similar size (see Schröter & Schroeder, 2017, for effect size estimates with the DWDS word frequency). This effect size reduction may be the result of the lower lexical richness of the best-fitting LLM corpora. The corpora with the highest lexical richness (childLex and DeepSeek) included nearly all words in the DeveL dataset (> 96%; see Figures B.3 and C.2). In contrast, the corpora with lower lexical richness included lower numbers of words from the DeveL selection of words. Thus, LLM word frequency was zero for more words in this subset compared to childLex or the DeepSeek corpora (see the right panel of Figure B.2). This distinction in the word frequency distribution of the DeveL words was also the most apparent difference between frequency measures. This finding indicates that children typically know these words less well, such that the word frequency estimate that excludes the word better represents the mental lexicon of children. Low-frequency or unknown words are important here, as words that are not yet or recently learned must be processed in a different way compared to already established words (e.g., see Gagl & Gregorová, 2024; Gagl, Richlan, et al., 2022). Even for adults, when an unknown word is encountered, a different behavior has to be established compared to when a known word is encountered (i.e., look up in a lexicon vs. move to the next word to establish sentence/text comprehension). This finding is also in line with investigations of children’s vocabulary that show an increase in vocabulary size with age (e.g., Keuleers et al., 2015; Segbers & Schroeder, 2017). Note that estimations of low-frequency words are inherently noisier than high-frequency words, as fewer observations are present.

Why does LLM word frequency result in a better fit to reading performance data? It is unlikely that LLMs closely mimic the mental representations of children, given the distinct nature of LLMs. Their training data is too large (Frank, 2023a), and the architectures are non-biologically plausible (Frank, 2023a). LLM word frequency could be a coincidental product of a regularization process applied to the word frequency in the original training data. LLMs’ computational mechanisms are designed to prevent overfitting and encourage the production of smoother, more generalizable words. Thus, generating words that occur slightly more often may be more appropriate in many contexts. Authors of children’s books, on the other hand, are motivated to select words based on literary interests (Korochkina & Rastle, 2025).

Oh et al. (2024) found that surprisal estimates from lower-performing models, e.g., as measured by perplexity (less predictive models), can better fit human reading times (see also Boeve & Bogaerts, 2024). Here, we found that children’s stories generated by Llama-3.3-70B-Instruct and GPT3.5-turbo are less lexically rich compared to DeepSeek-V3, but better explained the frequency effect in children’s reaction times. If we assume that lower lexical richness is a result of lower model performance in general, our results are consistent with this finding. Specifically, the models in our study that generate less lexically rich texts provide a better fit to lexical decision times. They, therefore, may better reflect an overlap of the vocabulary used by the model and stored in the children’s mental lexicon. It seems to be an empirical question, how lexical richness relates to model perplexity.

### Limitations and Future Directions

In this study, we focused on text targeted at German children and reading performance measured by lexical decision times from German children. It is unclear whether LLMs can generate linguistic corpora for other, less well-represented groups, languages, and reading behaviors (Blasi et al., 2022; Gagl, Gregorova, et al., 2022). As discussed above, more research is necessary to investigate the limitations of such extrapolation and the development of new resources that are currently unavailable but urgently needed (Blasi et al., 2022; Henrich et al., 2010). Extrapolating to groups underrepresented in the training data will likely be affected by the Western bias in currently used training data (Atari et al., 2023; Rystrøm et al., 2025). This is supported by results indicating that simply adding multilingual training data does not necessarily improve multilingual LLM performance, possibly due to capacity limits (Chang et al., 2023). The LLMs used here are not specifically developed for multilingual tasks and can achieve worse performance compared to specialized models in multilingual tasks (Lai et al., 2023). German can be considered a high-resource language that can outperform languages like Chinese or Vietnamese across several multilingual tasks (Lai et al., 2023). Thus, practical uses may conflict with current model biases, particularly for low-resource and non-Western languages. Although little is known about LLM performance in low-resource languages, the approach described here could be a first step. Recent investigations show that using language-specific LLMs can be beneficial for the extraction of psycholinguistic measures (Boeve & Bogaerts, 2024).

In Experiment 3, we replicated findings from Experiment 1 with open-weight models. A next step is to select less complex models, potentially in toy examples, with all parameters known. We initiated this study using a non-transparent, large, and continuously evolving model. Mechanistic explanations become impossible when complexity is so high that the model becomes a black box (Bender et al., 2021). For studying cognitively plausible ways in which word frequency approximates reading times, LLMs that are only as large as developmentally plausible are necessary (see Feng et al., 2024; Hu & Frank, 2024; Tan et al., 2024, for recent work in this direction). Furthermore, performance changes in unpredictable ways for non-transparent large language models (LLMs) that are being fine-tuned continuously using reinforcement learning with human feedback (RLHF Bai et al., 2022; Chung et al., 2024; Perez et al., 2022; Ziegler et al., 2020). In this process, LLMs are trained to better align with user requirements. This alternative optimization leads to a necessary trade-off between performance on next-word prediction and performance on alignment. Here, LLM word frequency from Experiment 1 resulted in much better model fits (AIC > 100) compared to the replication condition from Experiment 2. This difference is most likely due to changes between different snapshot versions of the same model that we used for Experiments 1 and 2.

**Figure B.8.**
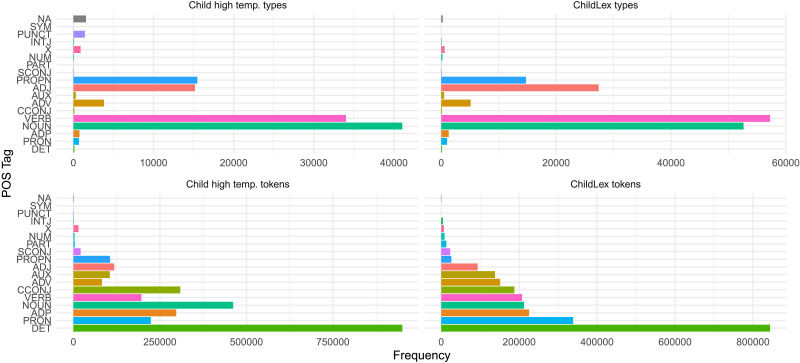
Distribution of POS tags in childLex and LLM corpora based on tokens (bottom panels) and types (top panels). The ordering of all panels follows the order of childLex tokens.

**Figure B.10.**
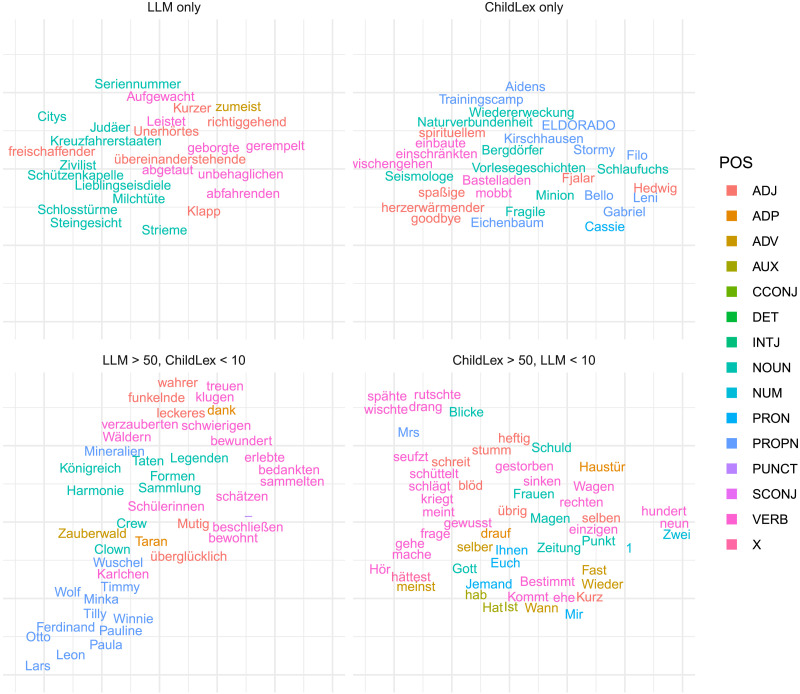
Distributions of a random sample of word embeddings using dimensionality reduction with UMAP to word vectors from FastText-German. Embeddings were reduced from 300-dimensional vectors. Color coding corresponds to different parts-of-speech (POS) tags as based on UDPipe’s “german-hdt” model. The figure shows four different selections of words based on their occurrence and frequency in childLex and ChHT.

With respect to corpus comparison, the lexical richness measures and word reading times we used here are simplistic qualities of text compared to the cultural, social, and ethical themes and pedagogical considerations underlying the text of children’s books (see e.g., Korochkina & Rastle, 2025). This study does not discuss the higher-level syntax or semantics of the books analyzed here (but see Figures B.8, B.9 for an analysis of POS and Figure B.10 for an analysis of word embeddings). Furthermore, the study does not provide a detailed examination of whether the LLM text can be used to evaluate these qualities. The text generated for this study is available (i.e., see OSF.io) and can still be used for such investigations. We showed that explained variation in word reading times depends on how lexical properties are quantified, including word frequency. We showed that LLM text contains atypical patterns, such as the spillover effects from English to German or numerous words found only in the LLM corpus but not in childLex. Such patterns suggest that generated text cannot substitute for human text, particularly in settings involving vulnerable participants (e.g., in the context of schools or the broader public).

LLM word frequency explains more variance, but results in a smaller effect size compared to child book-based word frequency. These findings are difficult to explain within the current approach. How can cognitively implausible LLM word frequency be more informative about processing measures than traditionally used book-based word frequency? This is an empirical question with many possible answers. Approaching an answer will involve studying how adults infer which words are known by children, how well they can infer children’s vocabulary, and if authors, on purpose, add less well-known words to their books for pedagogical purposes, e.g, to stimulate vocabulary growth (Korochkina & Rastle, 2025).

## CONCLUSIONS

Surprisingly, word frequency based on child-directed LLM text is similar to existing children’s book-based word frequency (e.g., childLex). Lexical richness seems to depend on the type of LLM. LLM word frequency better describes the effect of word frequency on reading performance in German children, but the estimated effect size is smaller. Thus, LLM corpora seem to open up new possibilities for investigating the word frequency effect, one of the strongest and most replicated effects in psycholinguistic research (Brysbaert et al., 2018), but also relevant to other domains of cognitive psychology (i.e., object recognition Gregorová et al., 2023).

Still, caution is advised when trying to understand the possibilities of LLMs for language development research. LLMs deviate in crucial ways from natural language acquisition pathways. The different nature of LLMs results, on the one hand, in surprisingly different patterns of language use, and on the other hand, in patterns of word processing cost that closely follow empirical data from children. The result is an approach to quantify and compare how the elements of LLM text correlate with metrics from classic corpora and human behavior.

## Funding Information

This research was supported by the University of Cologne and the German Research Fund (DFG, N* 523332674).

## Author Contributions

J.S.: Conceptualization; Methodology; Software; Writing – original draft; Writing – review & editing. H. W.: Software. N.M.: Conceptualization; Writing – review & editing. B.G.: Conceptualization; Methodology; Software; Writing – original draft, Writing – review & editing.

## Availability of Data, Materials, Code, and Supplementary Materials

See our OSF repository: osf.io.

## Figures and Tables

**Figure A.1.**
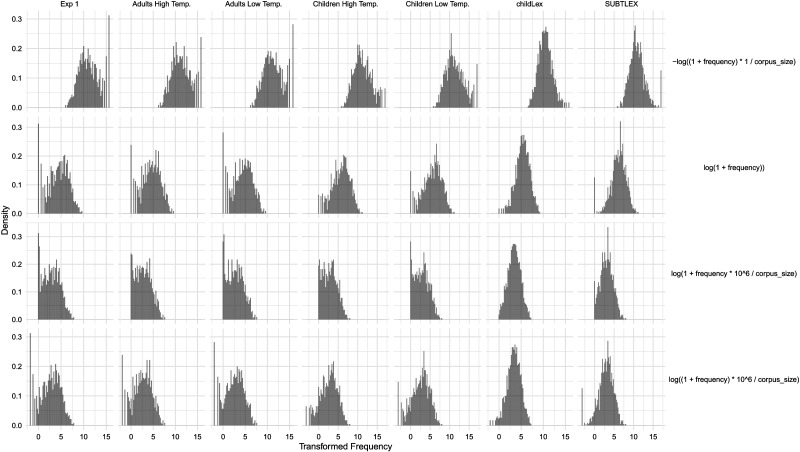
Histograms of transformed word frequency values. The *x*-axis shows the transformed word frequency values, and the *y*-axis shows the density of the values. The facets show the different transformations and the different corpora. The transformation in the third row results in a hard cut-off at 0 while the other 3 transformations result in more normal-like distributions.

**Figure A.2.**
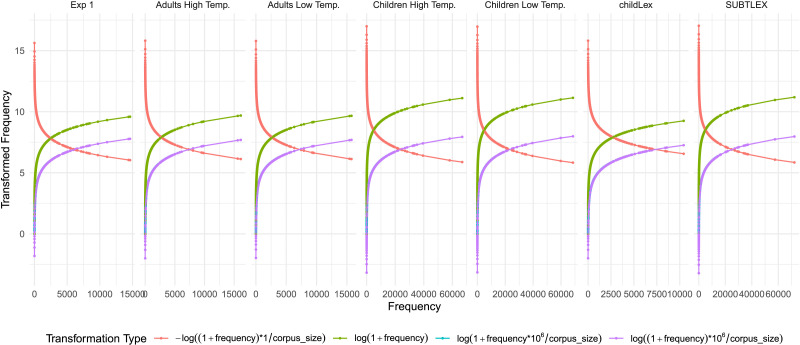
Scatter plots of transformed word frequency values. The facets show the different corpora.

**Figure A.3.**
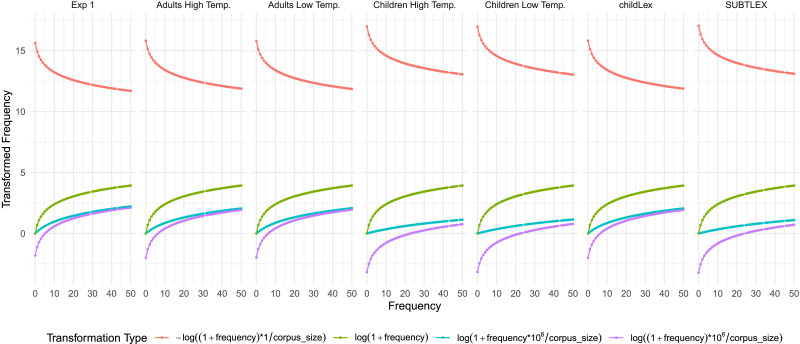
Zoomed in version of Figure A.2, showing only observed word frequencies smaller than 50.

**Figure A.5.**
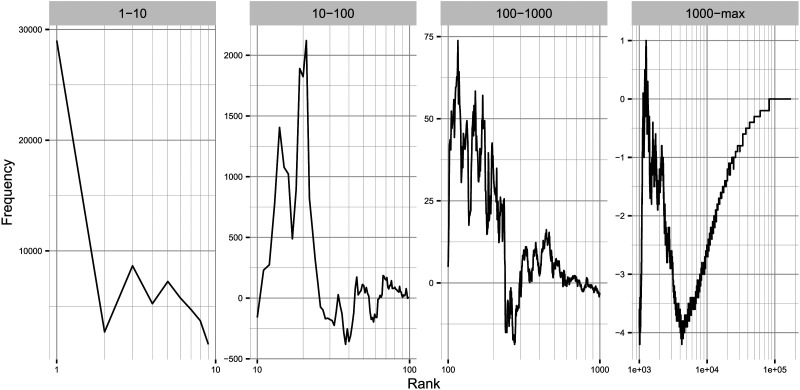
Differences between word frequency calculated based on the LLM and childLex corpora (i.e., the difference between the curves from Figure 2).

**Figure A.6.**
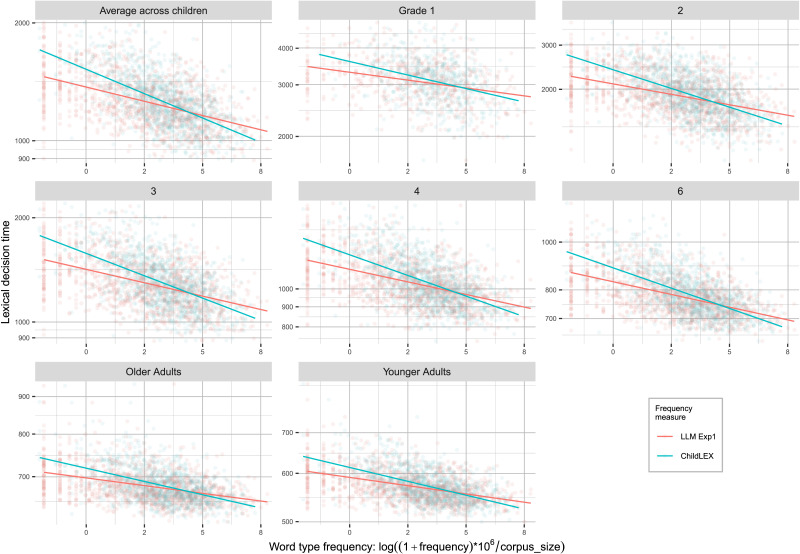
Relation between log reaction times (ms) with LLM and childLex log-frequency.

**Figure A.7.**
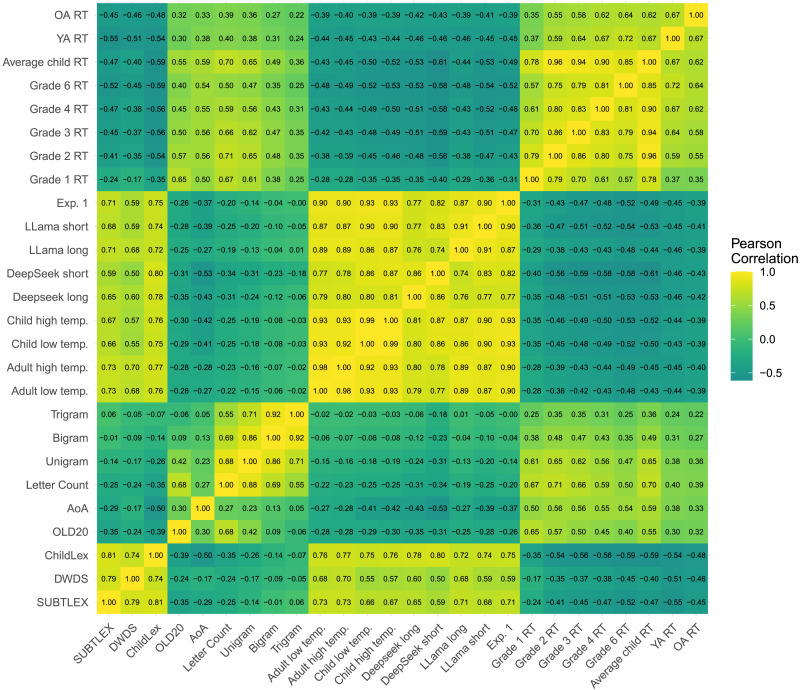
Correlation matrix for the Devel subset of words for which reaction times are available. The correlations with corpus-based word frequency measures are based on a $\log\left(\frac{\left(1+\text{frequency}\right)\times 10^{6}}{\text{corpus}\_\text{size}}\right)$ transformation. These corpora include: SUBTLEX, DWDS, childLex, as well as the LLM corpora.

**Figure A.8.**
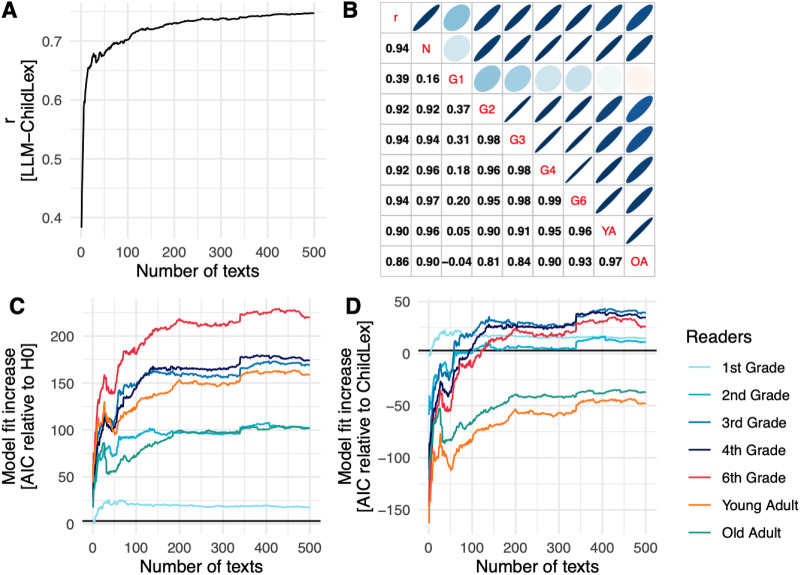
Partial LLM corpus analysis. (A) Subset-based estimation of the correlation between LLM type frequency and childLex type frequency (*y*-axis). We estimated the correlation 500 times based on an LLM word frequency extracted from a corpus that included only the first book. After that, we included all generated text from one additional book until all books were included. Note that this analysis was implemented for the words used in the DeveL dataset. (B) Pearson correlation matrix investigating the partial data curves from A and C. *r* represents the correlations from A; *N* represents the log-transformed number of stories (i.e., *x*-axis from A, C, or D); *G1-6*, represent the AIC differences from Grade 1 to 6 shown in C; *YA* and *OA* represent the younger and older adults AIC differences shown in C. Lower matrix shows the correlation coefficient *r* and the upper matrix the color coded (blue: positive correlation; white: no correlation; red: negative correlation) correlation silhouettes (narrow silhouettes indicate high and wide silhouettes indicate low correlations). (C) AIC differences for linear mixed models of the DeveL reaction times. The models included word frequency measures estimated on a subset of the LLM corpus (i.e., same subsets as in A), which were compared to the model without a word frequency measure included, and (D) when the baseline model included childLex word frequency.

**Figure B.5.**
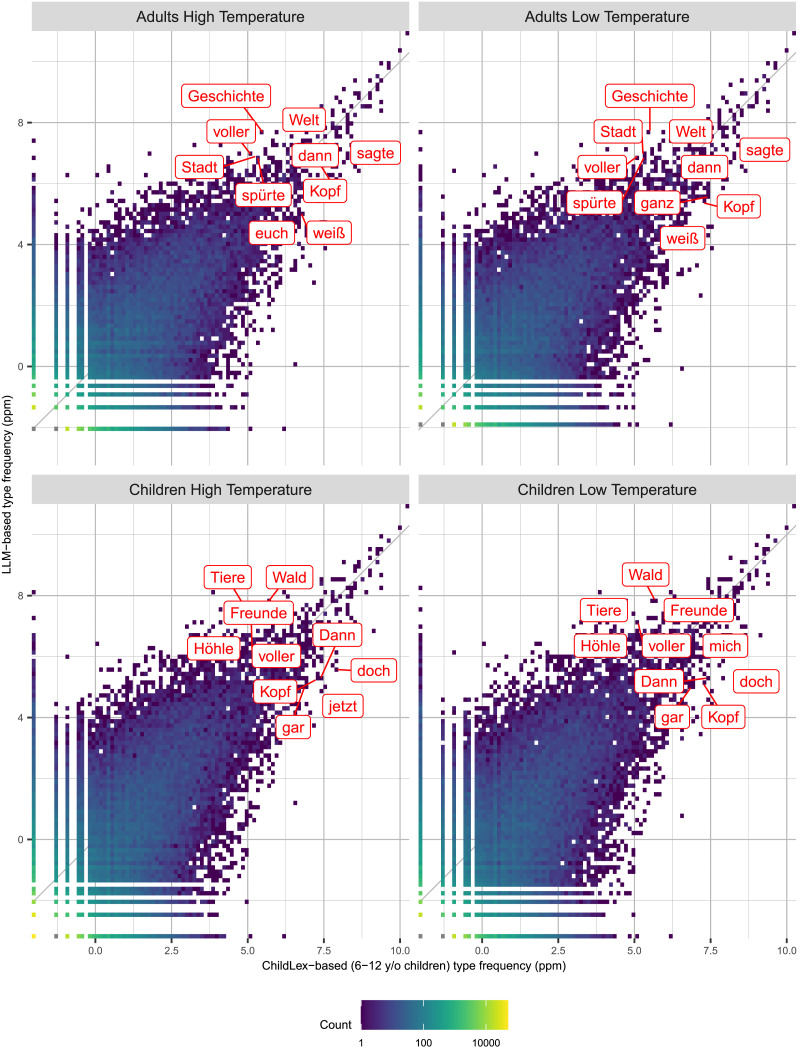
Scatterplots between LLM type frequency (*y*-axis) and childLex type frequency (*x*-axis; dark gray line) for all four corpora from Experiment 2. The labels show the top five differences on both sides (*x*-*y* and *y*-*x*). The color gradient of the dots represents the number of data points each dot represents.

**Figure B.6.**
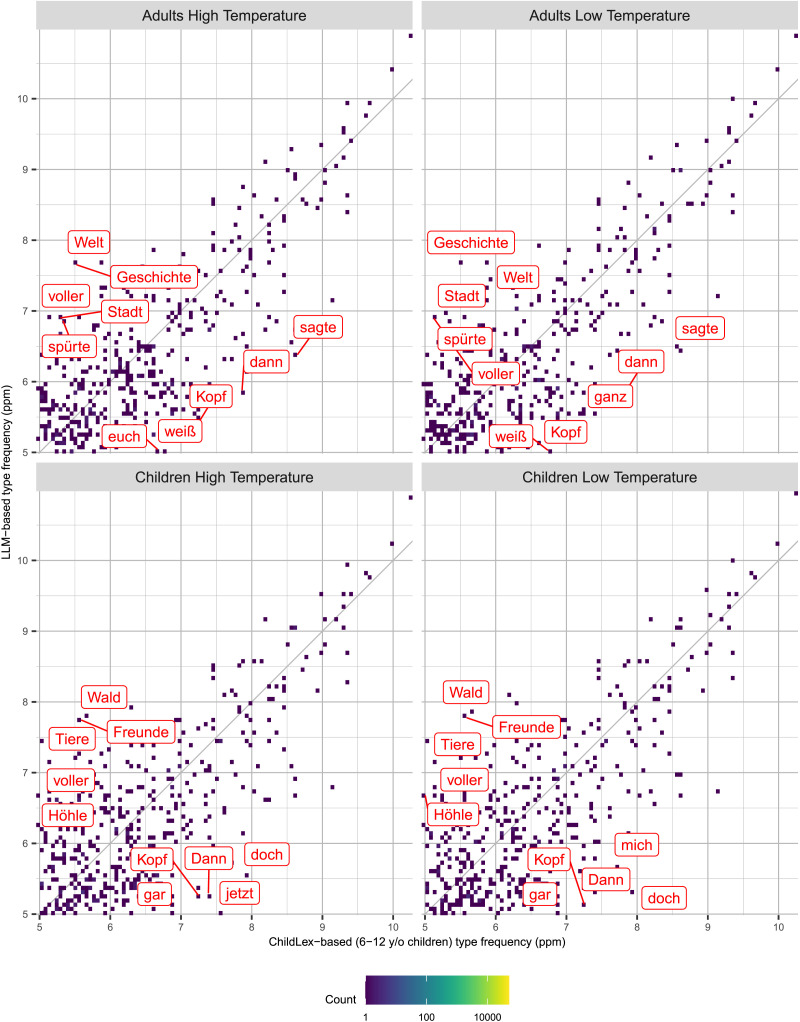
Zoomed in version of B.5.

**Figure B.7.**
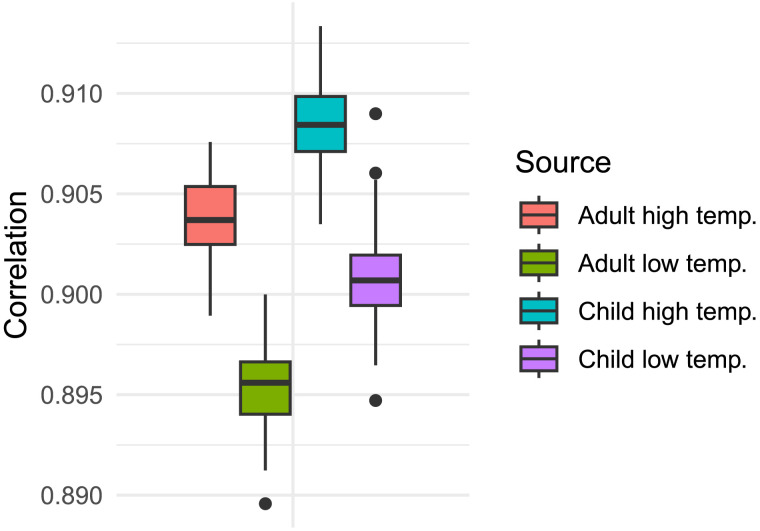
Split half reliability estimates. Box plots show 100 split-half Pearson correlations, indicating the reliability of the log. word frequency measurement for each of the newly generated corpora. Boxes and whiskers represent the distribution of the data, with the box spanning the interquartile range (IQR), which captures the middle 50% of the data, and the whiskers extend to 1.5 times the IQR, indicating variability outside the upper and lower quartiles. Outliers beyond this range are displayed as individual points.

**Figure B.9.**
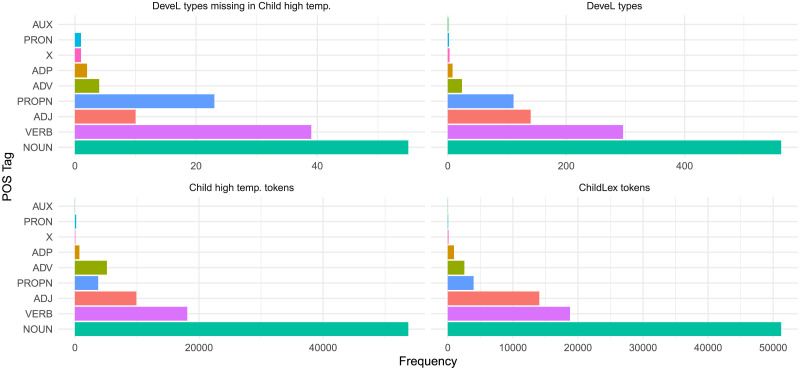
Distribution of POS tags for the subset of DeveL words that are also in the childLex and LLM corpora as based on tokens (bottom panels) and types (top panels). The ordering of all panels follows the order of childLex tokens again.

**Figure B.11.**
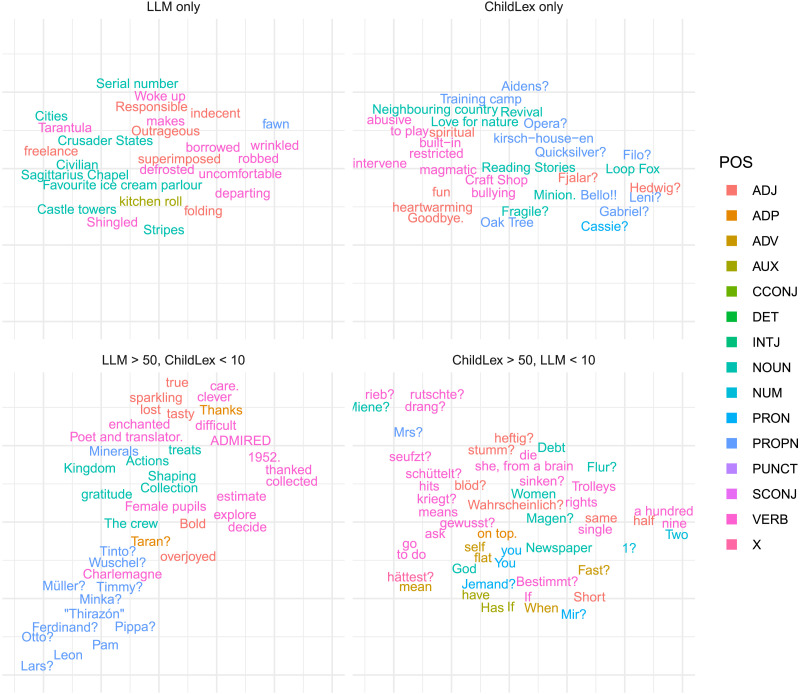
Translated version of Figure B.10. Words appearing with “?” indicate uncertainty, “!!” indicates translation failure. Some German words are not shown because of overlapping text labels.

## A: EXPERIMENT 1

### Word Frequency Transformation

The histograms in Figure A.1 show that

$$\log\left(\frac{\left(1+\text{frequency}\right)\times 10^{6}}{\text{corpus}\_\text{size}}\right),$$ (A.1)

is the most sensible transformation. Other options we considered included:

$$\log\left(1+\frac{\text{frequency}\times 10^{6}}{\text{corpus}\_\text{size}}\right),\;-\log\left(\frac{1+\text{frequency}}{\text{corpus}\_\text{size}}\right),\;\log\left(1+\text{frequency}\right)$$ (A.2)

The first transformation of Equation A.2 (third row in Figure A.1) results in a lower bound of 0 and an upper bound of 1 for normalized word frequency between 0 and 1. The resulting distribution collapses these values on this narrow interval, resulting in a skewed distribution.

Furthermore, the transformations in Figure A.2 show the effect of corpus size on the transformed word frequency. For larger corpora, which include the child-directed LLM corpora and SUBTLEX, the non-normalized transformation (log(1 + frequency)) results in the strongest differences acrosss the corpora.

Finally, the transformations in Figure A.3 finally show the different handling of low-frequency values. $\log\left(1+\frac{\text{frequency}\times 10^{6}}{\text{corpus}\_\text{size}}\right)$, behaves differently from the other 3 transformations by collapsing low word frequencies into a smaller range.

Note that log(1 + frequency) was the transformation used by e.g., SUBTLEX (Brysbaert et al., 2011). This transformation avoids negative values, and assigns 0 to missing words, similar to $\log\left(1+\frac{\text{frequency}\times 10^{6}}{\text{corpus}\_\text{size}}\right)$. However, the latter does not distinguish well enough between low-frequency words. Here, we are choosing a transformation ($\log\left(\frac{\left(1+\text{frequency}\right)\times 10^{6}}{\text{corpus}\_\text{size}}\right)$) that does normalize for corpus size, given the large differences we have here, see Figure A.2. The negative values also do not show big gaps between low and 0 word frequency values, see Figure A.3.

### Most Different Words

Table A.1 shows the top words from the one corpus that appear the least often in the other corpus.

**Table A.1.** The top frequent words that occur the least often in the other corpus, for all word lengths, and for words with more than 10 characters.

	childLex	childLex > 10	LLM	LLM > 10
1	Ganz	Wahrscheinlich	Jack	Sattelschlepper
2	Wieso	widersprach	Charaktere	Mäusepension
3	Wahrscheinlich	blitzschnell	Brownie	Schulgespenst
4	Bestimmt	Snorkfräulein	Brumm	aufgewecktes
5	Soll	verächtlich	Poppins	unvergessliches
6	verzog	irgendeinem	Zaubermaus	akzeptierten
7	kreischte	anschließend	Sattelschlepper	Korallenschatz
8	quer	irgendjemand	Fips	Abschlussfeier
9	presste	Zaubereiministerium	Hoppel	verantwortungsvoll
10	Meinst	Entschuldige	Mäusepension	unzertrennliche

### Litkey

We redrew the scatter plot from the main text using a corpus containing texts written by children themselves (Laarmann-Quante et al., 2019) instead of text written for children (Schroeder et al., 2015). This corpus is much smaller, but the resulting figure, see Figure A.4, shows the same pattern and the most differing words can be explained similarly as well.

### Rank Differences

Figure A.5 zooms in on the specific differences between childLex and LLM curves shown in Figure 2 from the main text, similar to Figure 2. Positive differences indicate a higher LLM word frequency, while negative differences indicate a lower LLM word frequency. Before rank 1,000, LLM words are roughly used more often, while after rank 1,000, LLM words are generally used less often. This finding again illustrates that the LLM uses high-frequency words more often and low-frequency words less often.

### Devel-Subset Correlation Matrix

Figure A.7 shows the correlation matrix for the subset of 1052 words from Devel (Schröter & Schroeder, 2017).

The correlations between Experiment 1 LLM word frequency and reaction time ranged between .31 (grade 1), to .52 (grade 6), while childLex word frequency correlated between .35 (grade 1) to .59 (grade 6), see Figure A.7. Both childLex and LLM word frequency correlated lower with adult reaction time (e.g., .48 and .39, respectively, for older adults). The LLM-childLex correlation was .75 for this subset of words, which is considerably larger than the correlation for the complete corpora (which was .69; see Figure 6).

The correlations for the Experiment 2 ChLT (the reproduced corpus) and the ChHT corpus were almost identical (maximally .02 difference). Both adult-directed corpora had lower correlations across all grades (.04–.07 lower). Correlations with adult reaction times were similar across the new conditions (maximally .01 difference for older adult reaction times). The correlations for the child-directed corpora also did not increase compared to Experiment 1 and were also comparable across conditions (maximally .02 difference), which we also found for the correlations between complete corpora.

For Experiment 3, we found the highest correlation between the average children’s RT’s and LLM-based word frequency for the DeepSeek short corpus (*r* = .61) compared to the other LLM word frequency measures (*r* between .53 and .43).

Together, the correlations show similar differences compared to the effect sizes obtained from regressions with covariates (see Figures 4 and 7).

### Effect Sizes

**Table A.2.** Effect size estimates per grade based on the complete set DeveL words.

	Estimate	SE	*t*	*p*	Adj. *R*^2^
Grade 1					
LLM Freq.	−27.1	6.1	−4.4	**< 0.001**	0.6363
childLex	−25.1	11.4	−2.2	**0.028**	0.6269
Grade 2					
LLM Freq.	−30.9	3.0	10.4	**< 0.001**	0.6916
childLex	−50.0	5.1	9.8	**< 0.001**	0.6886
Grade 3					
LLM Freq.	−24.4	1.8	13.6	**< 0.001**	0.6613
childLex	−37.0	3.1	11.8	**< 0.001**	0.6495
Grade 4					
LLM Freq.	−17.9	1.3	−13.8	**< 0.001**	0.5861
childLex	−27.7	2.3	−12.2	**< 0.001**	0.5734

### Effect Sizes

**Table A.3.** Effect size estimates per grade based on a subset of the DeveL data that excludes words with 0 occurrences in the LLM corpus.

	Estimate	SE	*t*	*p*	Adj. *R*^2^
Grade 1					
LLM Freq.	−33.7	7.3	4.6	**< 0.001**	0.6321
childLex	−22.1	12.0	1.8	0.066	0.6198
Grade 2					
LLM Freq.	−34.3	3.3	10.3	<**0.001**	0.6874
childLex	−48.1	5.3	9.1	**< 0.001**	0.681
Grade 3					
LLM Freq.	−25.6	2.0	12.7	<**0.001**	0.6562
childLex	−33.7	3.2	10.4	**< 0.001**	0.6404
Grade 4					
LLM Freq.	−19.6	1.5	13.3	**< 0.001**	0.5699
childLex	−26.3	2.4	11.1	**< 0.001**	0.55

### Robustness Analysis

To estimate the robustness of the correlation between LLM and childLex word frequency, we re-estimated LLM word frequency based on subsets from the complete corpus, specifically for the words used in the DeveL dataset (Schröter & Schroeder, 2017). For this analysis, we started with the first LLM texts generated based on a prompt using the first book. After that, we successively added the texts from the next book and re-estimated the correlations for each subset (see Figure A.8A). The curve shows a logarithmic increase in corpus similarity, indicating a substantial correlation increase of the two measures within the first 50 texts. After that, the increase in similarity is weaker, ending up at a correlation coefficient of just below .75. We tested whether the increase in similarity is indeed logarithmic. For this, we compared two correlations: The correlation between all correlation quotients (i.e., see *y*-axis in Figure A.8A) and (i) the numbers of texts (i.e., see *x*-axis in Figure A.8A) or (ii) the logarithm of the numbers of texts. The computed correlation for the linear number was lower (*r* = .71) than the correlation based on the logarithmic numbers (*r* = .94), indicating that the increase is indeed logarithmic (Figure A.8B also reports this correlation of .94 in the top left corner). The analysis shows that word frequency would not have been very different if we had used only half of the LLM corpus. It turns out that the correlation seems to keep increasing, but that it slows down with more text.

We also evaluated to what extent word frequency measures based on corpus subsets result in a lower model fit. Thus, we increased the corpus size (same as above), estimated the word frequency, and measured the model fit change when introducing the established word frequency measures compared to a model without the measure. We implemented this, starting with the texts from the first book. Ultimately, we can compare the absolute AIC difference values (i.e., AIC from the model with the new word frequency predictor minus the AIC based on the model without the predictor; the higher, the better the model fit) from all estimated word frequencies and all reading groups (1st–4th Grade, 6th Grade, younger and older adults; *N* tests = 500; see Figure A.8C). We rely on model comparison methods using linear regression models and the AIC, a measure optimal to investigate model fit change for newly introduced parameters. Note that a change in three AIC points is a significant model fit increase (i.e., the black horizontal line in Figure A.8C and D; all AICs above show a significant increase in model fit).

This partial corpus analysis shows that variation in reading performance can be explained better based on word frequency measures based on larger corpora (Figure A.8C), but that performance increases less, the larger the corpora. We find an increase in the AIC difference in all age groups, although the trend is very small in the youngest readers from Grade 1 (compare correlations of the size of the corpus - *N*- and Grade 1 - G1 - in contrast to the more older readers in Figure A.8B). Nonetheless, all analyses, except for the word frequency measures based on very small corpora (Number of books < 5) predicting the reaction times of Grade 1 readers, showed a significant increase in model fit. Correlations of AIC differences with increasing corpus size of all our groups of readers also showed that in Grade 1, the pattern differed from all other groups (Figure A.8B). In addition, groups with similar age (e.g., Grade 2 vs. Grade 3) had higher correlations (*r* range from .96 to .99) compared to comparisons with substantial age differences (e.g., Grade 2 vs. old adults; *r* = .81). Finally, the partial analysis that estimated the AIC differences against a baseline model including childLex word frequency as a predictor, showed that only linear models that predicted the reaction times of young readers (Grade 1–6) had an additional model fit increase based on the inclusion of LLM word frequency (see Figure A.8D). Models describing adult data did not benefit from introducing LLM word frequency. Also, we observed that for young readers, larger corpus sizes are needed for the word frequency measurement to produce a measure with higher descriptive power (Grade 1: 6 books; 2: 103; 3: 59; 4: 95; 6: 121; see Figure A.8D).

In the first analysis of reading performance, we found that the model fit of RTs increased when including a word frequency measure based on only a fraction of the full LLM corpus (*N* < 10 texts) for all age groups. Nonetheless, increasing the corpus size also increased the model fit further. For all but Grade 1 readers, the increase in model fit with a larger corpus was highly similar to the increase in the correlation between LLM- and childLex-based corpora described above (r range: .86–.94). The difference for Grade 1 was that model fit analysis peaked after about 10% of the corpus, with an incremental decrease of model fit for word frequencies based on larger corpora (i.e., when *N* > 100).

Similarly, we compared the model fit increase for LLM word frequency based on partial corpora to a model that already included childLex word frequency. Here, we investigated whether the new LLM word frequency explains variance over and above traditional word frequency measures. Most notably, this was the case for the Grade 1 readers after only a fraction of the texts included (*N* < 10). For Grades 2–6 readers, an increased model fit was found when the LLM corpus size was much larger (Grade 2: *N* > 100; Grade 3: *N* > 50; Grade 4: *N* > 100; Grade 6: *N* > 120). No additional variance was explained when childLex word frequency was already included for the two adult groups.

## B: EXPERIMENT 2

### Word Frequency Histograms

Figure B.2 shows histograms of word frequency distributions for all words as well as for the subset of DeveL words only. We included words from three LLM corpora as well as childLex. We grouped low word frequencies together to improve the readability of the plot.

The word frequency of the words in DeveL does not follow the natural distribution of word frequency as displayed in the left panel (i.e., a high number of low-frequency words and a low number of high-frequency words). Instead, the DeveL word frequency follows a normal distribution (when ignoring the lowest frequency cases), containing relatively many medium to high-frequency words. This strategy is reasonable for child experiments as one wants to sample from as many frequency ranges as possible in a limited number of trials, with words that can be expected to be known by young readers. Furthermore, the right panel shows that a relatively large number of DeveL words are not present in the LLM corpora; see the difference in the histogram at zero between the LLM and ChildLex corpora.

There are also general differences between the LLM and childLex corpora. Across all words, there are more childLex words with low frequencies, with a transition at around 20 words per million (at this point, the bars for childLex are not higher anymore). In the DeveL subset, there are more low LLM-based frequencies, with a transition at around 7 words per million. These difference indicate that the LLMs tends to use some different words that are not in the DeveL selection in places where it could have used DeveL word.

### Corpus Overlap

Figure B.3 shows overlap between the corpora, either for all words that were generated (left), or for the selection of words from the DeveL subset of words (right). Overlap with DeveL is almost 100%. Only four words from DeveL are missing in childLex (99.7% are overlapping), while 15 words are missing in ChHT (98.7%).

### Split Half Reliability

To estimate the reliability of the frequency measurement based on LLM corpora, we estimated the split-half reliability (see Figure B.7). We correlated LLM word frequency from a random selection of texts based on half of the book titles with the texts based on the other half of the book titles and repeated this 100 times. The correlations are thus based on correlating corpora that are based on completely different book titles with each other. Correlations range from an average of .895 for adults low temperature to .908 for the ChHT corpus (averaging across 100 random splits of 2 × 250 books). These correlations are higher compared to the LLM-childLex correlation, which was .62. The result, therefore, shows that the word frequency tables remain very similar across different book titles relative to childLex. Furthermore, correlations were higher in the high compared to the low temperature conditions, and they were higher in the child-directed compared to the adult-directed corpora, the latter likely due to the larger corpus size. Combined, this analysis showed that measuring word frequency based on LLM corpora is highly reliable overall.

### Distributions of POS Tags Across Types and Tokens

To get insight into the distribution of syntactal categories across corpora, we looked at the distribution of POS tags. We made use of UDPipe’s Universal Dependencies (UD) part-of-speech (POS) tagging scheme. Note that their NA tag is used when there is no clear category for a particular word.

Figure B.8 shows that LLM text contains a higher proportion of nouns vs verbs in both types and tokens compared to childLex. This indicates the use of more distinct nouns and more frequent noun use. In addition, childLex also has relatively more distinct adjectives and also more frequent adjective use. Thus, the increased lexical richness in childLex seems to be mostly due to the use of more distinct adjectives and verbs. The differences in the numbers of noun tokens suggest that LLM text is less action-focused and more descriptive.

### Distributions of POS Tags Across Types and Tokesn for the Devel Subset of Words Only

Figure B.9 shows that the distributions of tokens are largely similar, indicating the similarity between LLM and childLex frequency for the DeveL subset of words. The largest difference seems to be related to use of adverbs and adjectives. The top right panel shows what word types are included in DeveL and the top left panel shows which of these word types have a distinctively low frequency in the ChHT corpus. Note that UDPipe misclassified many of the proper nouns as nouns, so the blue bar is not necessarily informative. Thus, the distribution of DeveL types that are missing in the ChHT corpus does not seem very different from the actual distribution of types in Devel. For specific details about the selection criteria of DeveL, see the original DeveL paper (Schröter & Schroeder, 2017).

### Distributions of Word Embeddings After Dimension Reduction

Figure B.10 illustrates some syntactical and semantic differences between the corpora. For example, it shows that the distribution of word embeddings of words that are more prevalent in childLex includes a separate semantic area with mostly verbs (see bottom right corner in the bottom right panel) while words that are more prevalent to the LLM corpus (we selected the ChHT corpus for this analysis) includes a separate area with mostly proper nouns.

### Distributions of Word Embeddings After Dimension Reduction (Translated)

Figure B.11 shows a translated version of Figure B.10. German words were translated to English using the MyMemory API (mymemory.translated.net), a free translation service based partly on human-translated text segments.

### Words Not in ChildLex or LLM Corpus

Tables B.2, B.1, B.4, and B.3 show the top frequent words that occur in only one of both corpora, for all word lengths, and for words with more than 10 characters.

**Table B.1.** Words not in Childlex or in the AdLT corpus

childLex	F	childLex > 10	F	AdLT	F	AdLT > 10	F
daβ	6.1	widersprach	3.6	Max	7.6	Charakteren	4.5
1	5.1	SternenClan	3.5	Anna	7.1	Schulvampire	4.4
Wieso	4.8	Olchi-Kinder	3.5	Lena	6.5	einzustehen	4.3
Gleich	4.4	kopfschüttelnd	3.1	Lisa	6.4	nahegelegenen	4.3
guckte	4.3	vorwurfsvoll	3.0	Müller	6.3	thematisiert	4.2
Bestimmt	4.3	Viertelstunde	3.0	Mia	6.1	unvergessliche	4.1
mußte	4.2	augenblicklich	2.9	Emma	5.8	kindgerechte	4.0
Jedenfalls	4.2	Mottenflügel	2.9	Paul	5.7	einfühlsame	3.9
guckt	4.2	Anscheinend	2.9	Harry	5.7	Protagonisten	3.9
kriegt	4.2	stirnrunzelnd	2.9	Julia	5.6	faszinierendes	3.9

**Table B.2.** Words not in Childlex or in the AdHT corpus

childLex	F	childLex > 10	F	AdHT	F	AdHT > 10	F
daβ	6.1	SternenClan	3.5	Max	7.3	Charakteren	4.6
1	5.1	Olchi-Kinder	3.5	Anna	6.9	nahegelegenen	4.3
guckte	4.3	einigermaßen	3.0	Lena	6.3	einzustehen	4.2
Bestimmt	4.3	Viertelstunde	3.0	Lisa	6.1	unvergessliche	4.1
Blattsee	4.2	Mottenflügel	2.9	Mia	6.1	thematisiert	4.1
mußte	4.2	stirnrunzelnd	2.9	Müller	6.1	Schulvampire	4.1
Jedenfalls	4.2	Wolkenpfote	2.8	Paul	5.8	Protagonisten	4.0
guckt	4.2	ausnahmsweise	2.8	Emma	5.8	kindgerechte	3.9
kriegt	4.2	Ausgerechnet	2.7	Julia	5.7	herzerwärmende	3.8
Dad	4.2	verschränkte	2.7	Harry	5.6	faszinierendes	3.7

**Table B.3.** Words not in Childlex or in the ChLT corpus

childLex	F	childLex > 10	F	ChLT	F	ChLT > 10	F
daβ	6.1	Olchi-Kinder	3.5	Max	8.3	nahegelegenen	5.0
1	5.1	Wohnungstür	3.1	Mia	7.5	Schulvampire	4.5
jedenfalls	4.8	einigermaßen	3.0	Lina	7.0	unvergessliche	4.2
guckte	4.3	Viertelstunde	3.0	Lena	6.7	Sternenfohlen	4.1
mußte	4.2	Mottenflügel	2.9	Emma	6.6	einzustehen	4.1
Jedenfalls	4.2	stirnrunzelnd	2.9	Tim	6.5	Inselschüler	4.0
kriegt	4.2	entgeistert	2.8	Felix	6.4	Schafgäääng	3.9
muβ	4.1	Wolkenpfote	2.8	Paul	6.3	KuchenMonster	3.9
0	4.1	Unglaublich	2.8	Lilli	6.2	SkaterBande	3.7
wär	4.1	ausnahmsweise	2.8	Finn	6.1	abenteuerlustiger	3.5

**Table B.4.** Words not in Childlex or in the ChHT corpus

childLex	F	childLex > 10	F	ChHT	F	ChHT > 10	F
daβ	6.1	Olchi-Kinder	3.5	Max	8.1	nahegelegenen	4.9
1	5.1	unwillkürlich	3.1	Mia	7.3	Schulvampire	4.4
mußte	4.2	Wohnungstür	3.1	Lina	6.7	unvergessliche	4.2
Jedenfalls	4.2	Viertelstunde	3.0	Lena	6.6	Sternenfohlen	4.1
kriegt	4.2	Mottenflügel	2.9	Emma	6.6	Inselschüler	4.0
muβ	4.1	stirnrunzelnd	2.9	Tim	6.6	einzustehen	4.0
0	4.1	ausnahmsweise	2.8	Felix	6.4	Schafgäääng	3.8
wär	4.1	Ausgerechnet	2.7	Paul	6.3	KuchenMonster	3.8
andern	4.1	verständnislos	2.7	Finn	6.2	SkaterBande	3.7
wußte	3.9	Premierminister	2.7	Anna	6.2	abenteuerlustiger	3.4

### Top Frequency Words Occurring the Least Often in ChildLex or LLM Corpus

Tables B.6, B.5, B.8, and B.7 show the top frequent words that occur the least often in the other corpus, for all word lengths, and for words with more than 10 characters.

**Table B.5.** The top frequent words that occur the least often in the other corpus, for all word lengths, and for words with more than 10 characters for the AdLT corpus

childLex	F	childLex > 10	F	AdLT	F	AdLT > 10	F
wenigstens	4.9	Hoffentlich	4.4	unterhaltsame	5.6	unterhaltsame	5.6
Eigentlich	4.7	Schnupferich	3.6	lehrreiche	5.0	unschlagbares	4.3
Hoffentlich	4.4	Zehenspitzen	3.3	Charaktere	5.0	Perspektiven	3.6
vorhin	4.2	verächtlich	3.1	Bindung	4.9	Korallenschatz	3.5
irgendwas	4.1	einigermaßen	3.0	Teamwork	4.5	vielfältige	3.4
blöd	4.1	Pfannkuchen	2.9	unschlagbares	4.3	Sattelschlepper	3.4
kreischte	3.9	entgeistert	2.8	humorvolle	4.0	unvergesslicher	3.4
Mist	3.8	Ausgerechnet	2.7	Jack	3.9	weiterzuentwickeln	3.4
Mehr	3.8	Mittlerweile	2.7	zeitlose	3.8	Mäusepension	3.3
Klo	3.8	verschränkte	2.7	Überwinden	3.8	authentisch	3.3

**Table B.6.** The top frequent words that occur the least often in the other corpus, for all word lengths, and for words with more than 10 characters for the AdHT corpus

childLex	F	childLex > 10	F	AdHT	F	AdHT > 10	F
jedenfalls	4.8	Wahrscheinlich	4.6	unterhaltsame	5.4	unterhaltsame	5.4
Wahrscheinlich	4.6	Hoffentlich	4.4	Charaktere	5.0	unschlagbares	4.0
flüstert	4.4	Unglaublich	2.8	lehrreiche	4.8	Perspektiven	3.7
Hoffentlich	4.4	verständnislos	2.7	Bindung	4.8	vielfältige	3.6
Quatsch	4.1	Premierminister	2.7	Teamwork	4.3	Mäusepension	3.5
hockte	4.0	Seidenschnabel	2.6	humorvolle	4.1	Sattelschlepper	3.3
Dem	3.9	Offensichtlich	2.6	unschlagbares	4.0	unzertrennliche	3.3
Ihm	3.8	Erstklässler	2.6	zeitlose	3.9	Bedrohungen	3.3
Meinst	3.8	Großmutters	2.6	Ermittler	3.8	vielschichtigen	3.3
gucken	3.8	irgendwelchen	2.5	Poppins	3.7	Korallenschatz	3.3

**Table B.7.** The top frequent words that occur the least often in the other corpus, for all word lengths, and for words with more than 10 characters for the ChLT corpus

childLex	F	childLex > 10	F	ChLT	F	ChLT > 10	F
wenigstens	4.9	Augenbrauen	3.7	unschlagbares	4.6	unschlagbares	4.6
hockte	4.0	SternenClan	3.5	Brumm	4.2	unzertrennliche	3.9
Dem	3.9	unwillkürlich	3.1	unzertrennliche	3.9	Sattelschlepper	3.8
Ihm	3.8	augenblicklich	2.9	Sattelschlepper	3.8	unvergessliches	3.8
Augenbrauen	3.7	Treppenhaus	2.9	unvergessliches	3.8	aufgewecktes	3.6
Immerhin	3.7	Einverstanden	2.7	Charaktere	3.8	Mäusepension	3.6
nachher	3.6	Vorbeigehen	2.6	Poppins	3.8	unvergesslicher	3.5
Deswegen	3.6	Offensichtlich	2.6	Holle	3.8	Korallenschatz	3.5
Außer	3.5	Krankenflügel	2.6	aufgewecktes	3.6	Schulgespenst	3.4
SternenClan	3.5	irgendwelchen	2.5	Zaubermaus	3.6	unterhaltsame	3.3

**Table B.8.** The top frequent words that occur the least often in the other corpus, for all word lengths, and for words with more than 10 characters for the ChHT corpus

childLex	F	childLex > 10	F	ChHT	F	ChHT > 10	F
Neunauge	4.0	Anscheinend	2.9	unschlagbares	4.5	unschlagbares	4.5
Meinst	3.8	entgeistert	2.8	Brumm	4.0	unzertrennliche	3.9
Mehr	3.8	Mittlerweile	2.7	unzertrennliche	3.9	unvergessliches	3.8
Madam	3.7	verschränkte	2.7	Charaktere	3.8	Sattelschlepper	3.7
Mum	3.6	Großmutters	2.6	Poppins	3.8	aufgewecktes	3.6
womöglich	3.5	gerunzelter	2.5	unvergessliches	3.8	unvergesslicher	3.5
Außer	3.5	vorsichtshalber	2.5	Sattelschlepper	3.7	Schulgespenst	3.5
gekriegt	3.5	umständlich	2.3	Holle	3.7	Mäusepension	3.5
Wozu	3.5	Donnerwetter	2.3	Bindung	3.6	Korallenschatz	3.5
werd	3.4	unvermittelt	2.3	aufgewecktes	3.6	unterhaltsame	3.3

## C: EXPERIMENT 3

### Corpus Overlap

Figure C.2 shows overlap between the Experiment 3 corpora, similar to Figure B.3. The left panel shows overlap for all generated words and the right panels shows overlap for the selection of DeveL words only. Overlap with DeveL is almost 100%. 14 DeveL words are missing in DeepSeek long (98.7%). For example, the DeepSeek long corpus did not contain words like Apfelsine, Automatik, Fasching (out-of-fashion words for orange, automatic, and Carnival respectively).

### Lexical Richness

Predicted number of types in hypothetical 10-million-token corpora.

### Words Not in ChildLex or LLM Corpus

Tables C.1, C.2, C.3, and C.4 show the top frequent words that occur in only one of both corpora, for all word lengths, and for words with more than 10 characters.

**Table C.1.** Words not in Childlex or in the DS-long corpus

childLex	F	childLex > 10	F	DS-long	F	DS-long > 10	F
1	5.1	Brombeerkralle	3.8	Lina	7.6	Protagonist	4.3
Blattsee	4.2	Olchi-Kinder	3.5	Max	6.9	Protagonistin	4.2
mußte	4.2	einigermaßen	3.0	Lena	6.7	ausarbeiten	3.7
muß	4.1	Anschließend	3.0	Tom	6.4	Protagonisten	3.6
0	4.1	Anscheinend	2.9	Mia	6.3	Algorithmus	3.3
Neunauge	4.0	ausnahmsweise	2.8	Oh	6.3	Algorithmen	3.2
Brombeerkralle	3.8	Mittlerweile	2.7	Ben	6.2	Postskriptum	3.2
T-Shirt	3.5	verständnislos	2.7	Leo	6.2	Nebenfiguren	3.1
Olchi-Kinder	3.5	Premierminister	2.7	Finn	6.0	inspirierte	3.0
bißchen	3.4	Tagespropheten	2.7	Emma	5.9	einzustehen	3.0

**Table C.2.** Words not in Childlex or in the DS-Short corpus

childLex	F	childLex > 10	F	DS-Short	F	DS-Short > 10	F
daß	6.1	Brombeerkralle	3.8	Lina	8.7	Leseanfänger	4.9
1	5.1	Olchi-Kinder	3.5	Lena	7.9	Mississippi	4.0
allmählich	4.3	einigermaßen	3.0	Max	7.8	Selberlesen	4.0
Blattsee	4.2	Viertelstunde	3.0	Ben	7.6	Schmuddelfing	3.9
mußte	4.2	Anschließend	3.0	Tom	7.5	selbstgebastelten	3.8
muß	4.1	Mottenflügel	2.9	Finn	7.1	Scherzfragen	3.2
0	4.1	eindringlich	2.9	Leo	7.0	KuchenMonster	3.2
Neunauge	4.0	Anscheinend	2.9	Mia	6.9	SkaterBande	3.1
wußte	3.9	stirnrunzelnd	2.9	Paul	6.4	ParkSheriffs	3.1
Brombeerkralle	3.8	entgeistert	2.8	Tim	6.3	Sonnenschule	3.1

**Table C.3.** Words not in Childlex or in the Llama-long corpus

childLex	F	childLex > 10	F	Llama-long	F	Llama-long > 10	F
daß	6.1	Wahrscheinlich	4.6	Tim	7.7	Selbstreflexion	5.2
eben	5.8	Hoffentlich	4.4	Max	7.6	inspirierte	4.9
Ganz	5.1	Taschenbier	4.0	Lena	7.3	Initiativen	4.9
1	5.1	ausgerechnet	4.0	Müller	6.4	Nachhaltigkeit	4.8
sowieso	5.0	Brombeerkralle	3.8	Timmy	6.3	Überzeugungen	4.4
allerdings	5.0	Hosentasche	3.7	Leo	6.2	Empfehlungen	4.3
selber	5.0	Schnupferich	3.6	Emma	6.0	kulturellen	4.3
Eigentlich	4.7	anscheinend	3.5	Harry	5.5	nachhaltigen	4.2
Wahrscheinlich	4.6	Zeigefinger	3.5	Epilog	5.4	unvergessliche	4.1
Stimmt	4.5	SternenClan	3.5	Anna	5.4	inspirierten	4.0

**Table C.4.** Words not in Childlex or in the Llama-short corpus

childLex	F	childLex > 10	F	Llama-short	F	Llama-short > 10	F
daß	6.1	Wahrscheinlich	4.6	Max	8.5	nahegelegenen	5.0
Nun	5.9	Taschenbier	4.0	Tim	8.3	ParkSheriffs	4.1
Ganz	5.1	Brombeerkralle	3.8	Lena	8.2	unvergessliche	4.0
1	5.1	Hosentasche	3.7	Leo	7.1	Schulvampire	3.9
allerdings	5.0	Schnupferich	3.6	Müller	6.9	KuchenMonster	3.9
offenbar	5.0	anscheinend	3.5	Emma	6.8	Inselschüler	3.9
selber	5.0	Zeigefinger	3.5	Timmy	6.7	PicknickEssen	3.8
wenigstens	4.9	SternenClan	3.5	Felix	6.5	abenteuerlustiger	3.8
deutete	4.9	mittlerweile	3.5	Anna	6.1	Schafgäääng	3.8
rasch	4.9	Olchi-Kinder	3.5	Lilli	6.1	Mississippi	3.7

### Top Frequency Words Occurring the Least Often in ChildLex or LLM Corpus

Tables C.5, C.6, C.7, and C.8 show the top frequent words that occur the least often in the other corpus, for all word lengths, and for words with more than 10 characters.

**Table C.5.** The top frequent words that occur the least often in the other corpus, for all word lengths, and for words with more than 10 characters for the DS-long corpus

childLex	F	childLex > 10	F	DS-long	F	DS-long > 10	F
daß	6.1	Viertelstunde	3.0	Twist	5.0	vergessenes	4.3
wußte	3.9	entgeistert	2.8	Optionen	4.7	flüsternden	3.7
erkundigte	3.7	Kinderstube	2.6	Charaktere	4.4	Menschlichkeit	3.6
Feuerstern	3.6	Einzelheiten	2.5	vergessenes	4.3	weiterspinnen	3.3
wisperte	3.5	Treppenabsatz	2.5	Gröβerem	4.1	Erweiterung	3.1
Hemul	3.1	hergekommen	2.4	Nachhall	3.9	Vergessenen	3.1
Daran	3.1	Detektivtagebuch	2.3	Teamwork	3.8	Perspektiven	3.0
Viertelstunde	3.0	Vorderpfoten	2.3	Fokus	3.8	Koordinaten	3.0
Allmählich	3.0	Umkleideraum	2.2	flüsternden	3.7	mysteriöser	3.0
sogleich	2.9	auszumachen	2.2	Dialogen	3.7	Gemeinschaften	2.9

**Table C.6.** The top frequent words that occur the least often in the other corpus, for all word lengths, and for words with more than 10 characters for the DS-Short corpus

childLex	F	childLex > 10	F	DS-Short	F	DS-Short > 10	F
selber	5.0	Staubfinger	3.3	Schneider	4.9	Detektivgeschichte	3.5
andern	4.1	aufgebracht	3.1	Teamwork	4.3	überlisteten	3.3
höchst	3.5	kopfschüttelnd	3.1	Brumm	4.1	flüsternden	3.3
Staubfinger	3.3	Wohnungstür	3.1	Weber	4.0	Mäusepension	3.1
weshalb	3.3	Angelegenheit	3.0	Hoppel	4.0	Herbstabend	3.1
Hastig	3.3	augenblicklich	2.9	Poppins	3.8	Sattelschlepper	3.0
Wenigstens	3.2	ausnahmsweise	2.8	Detektivgeschichte	3.5	mysteriöser	3.0
Vermutlich	3.2	Tagespropheten	2.7	Holle	3.5	Kristallhöhle	2.9
Charlotte	3.2	möglicherweise	2.7	Kitz	3.4	regnerischer	2.9
aufgebracht	3.1	Vorbeigehen	2.6	überlisteten	3.3	Superkicker	2.9

**Table C.7.** The top frequent words that occur the least often in the other corpus, for all word lengths, and for words with more than 10 characters for the Llama-short corpus

childLex	F	childLex > 10	F	Llama-short	F	Llama-short > 10	F
eben	5.8	Augenbrauen	3.7	Jack	4.8	unzertrennliche	3.9
Eigentlich	4.7	widersprach	3.6	Brumm	4.4	Mäusepension	3.7
beinahe	4.4	blitzschnell	3.4	Poppins	4.2	unvergessliches	3.5
außerdem	4.4	aufgebracht	3.1	Holle	4.1	Sattelschlepper	3.5
glaub	4.4	Gleichzeitig	3.1	Zaubermaus	4.0	unvergesslicher	3.4
weder	4.3	erstaunlich	3.0	unzertrennliche	3.9	verantwortungsvoll	3.4
ungefähr	4.3	Angelegenheit	3.0	Benz	3.9	Piratenschreck	3.3
guckt	4.2	umklammerte	3.0	Ravensburg	3.8	Wunschzauber	3.3
furchtbar	4.2	Jackentasche	3.0	Cleverness	3.8	abenteuerlustiges	3.2
Dad	4.2	hintereinander	2.8	Erinner	3.8	Geheimschwein	3.1

**Table C.8.** The top frequent words that occur the least often in the other corpus, for all word lengths, and for words with more than 10 characters for the Llama-long corpus

childLex	F	childLex > 10	F	Llama-long	F	Llama-long > 10	F
wenigstens	4.9	durcheinander	4.0	nachhaltige	5.5	nachhaltige	5.5
jedenfalls	4.8	unauffällig	3.3	Motivation	5.2	Gemeinschaften	4.9
Wieso	4.8	Normalerweise	3.3	Gemeinschaften	4.9	Technologien	4.6
zischte	4.7	protestierte	3.2	lokalen	4.8	Perspektiven	4.6
Klar	4.5	verächtlich	3.1	Ära	4.8	Selbstlosigkeit	4.2
schreit	4.4	irgendeinem	3.1	Jack	4.6	unterstützende	4.1
Dafür	4.3	unwillkürlich	3.1	Technologien	4.6	Unterstützen	3.9
Kaum	4.3	Treppenhaus	2.9	Perspektiven	4.6	weiterzuentwickeln	3.8
guckt	4.2	Schrottplatz	2.9	Charaktere	4.6	authentisch	3.7
Dad	4.2	schlagartig	2.9	Bindung	4.5	Zukunftsaussichten	3.6
